# A global analysis of plant nutrient limitation affected by atmospheric nitrogen and phosphorous deposition

**DOI:** 10.3389/fpls.2024.1473493

**Published:** 2024-12-19

**Authors:** Lan Du, Lisong Tang, Xinjun Zheng, Yan Li

**Affiliations:** ^1^ College of Ecology and Environment, Xinjiang University, Urumqi, China; ^2^ State Key Laboratory of Desert and Oasis Ecology, Xinjiang Institute of Ecology and Geography, Chinese Academy of Sciences, Urumqi, Xinjiang, China; ^3^ Fukang Station of Desert Ecology, Chinese Academy of Sciences, Fukang, Xinjiang, China; ^4^ State Key Laboratory of Subtropical Silviculture, Zhejiang A and F University, Hangzhou, Zhejiang, China

**Keywords:** N deposition, P deposition, N:P threshold hypothesis, threshold effect, net primary productivity, ecosystem P limitation

## Abstract

Uncovering the response of plant functional types (PFTs) to nutrient limitation caused by atmospheric deposition is critical for assessing the health of terrestrial ecosystems under climate change conditions. However, it remains unclear how atmospheric deposition and underlying ecological factors affect PFTs globally. To address this, we compiled a global dataset of four PFTs, i.e., herb, evergreen broad-leaf (EB), deciduous broad-leaf (DB), and conifer (CO), and utilized both linear mixed-effects models and structural equation models to describe the thresholds of their net primary productivity (NPP), and tested the relationships between their NPP and potential environmental drivers based on the N/P threshold hypothesis. We found that atmospheric N and P deposition non-linearly affected NPP and the effects were most pronounced for the EB, DB, and CO categories, with tipping points in the ranges of 8.32–9.33 kg N·ha^−1^·yr^−1^ and 0.20–0.30 kg P·ha^−1^·yr^−1^, respectively. Atmospheric N and P deposition negatively affected the NPP of approximately 53.68% and 43.88% of terrestrial ecosystem plants, respectively, suggesting increased P limitation and N saturation in most terrestrial ecosystems worldwide. We further determined that the N/P threshold hypothesis is applicable in assessing the effects of atmospheric N and P deposition on the growth of woody plants (EB, DB, and CO) through nutrient limitation. The results of this study will contribute to more effective landscape management in changing environments.

## 1 Introduction

Ensuring sustainable increases in net primary productivity (NPP) is a top priority in policy formulation worldwide (Duarte et al., 2022), because the loss of forests disrupts the ecosystem essential to human well-being (Wang et al., 2021). However, an increase in the NPP of terrestrial ecosystems requires large amounts of nutrients, mainly nitrogen (N) and phosphorus (P), to meet the stoichiometric needs of plant growth (Zhao et al., 2024). N and P, alone or in combination, limit NPP in most terrestrial ecosystems (Luo et al., 2021). Understanding how NPP responds to N and P limitations is crucial from both ecological and social perspectives.

Studies have indicated that the overall N/P ratio is not static and may vary with the environment (Rivas-Ubach et al., 2012; Sun et al., 2019). For example, an increase in temperature and water availability raise community-level N/P ratios (Fan et al., 2016) because of relatively more favorable growth conditions (Sun et al., 2017). However, under drought conditions, the N/P ratios might shift to improve the water-use efficiency (Peñuelas et al., 2020; Sardans et al., 2021b). Based on a global leaf N and P contents database (12,055 records), Tian et al. (2018b) questioned the 2/3-power law of scaling leaf N to P (N = *β*P*^α^*, where both *α* and *β* are constants) and revealed that *α* varies considerably between stations, latitude zones (e.g., tropical versus temperate), continents, and plant functional types (PFTs). This variation may be regulated by plant growth rate, soil N or P availability, leaf lifespan, and climate (Tian et al., 2018b). In addition, fertilization experiments with *Arabidopsis thaliana* have shown that the N/P relationship in plants is regulated by N and P nutrient availability (Yan et al., 2018). Moreover, at a global scale, as the input of N exceeds that of P in most ecosystems, the biosphere may shift from N-limited to P-limited or N/P co-limited conditions (Du et al., 2020). Nutrient imbalances between N and P may decrease ecosystem carbon (C) retention capacity (Chen and Chen, 2021) and affect ecosystem species composition, structure, diversity, and function (Peñuelas et al., 2020; Sardans et al., 2021b).

Although the N/P ratios of plants vary with the environment, changes within a certain range do not result in changes in nutrient restriction status or plant growth (Liu et al., 2020). For example, the N/P threshold hypothesis suggests that a specific N/P threshold determines the nutrient limitation of plant growth, serving as an indicator of spatiotemporal variations in plant physiological and ecosystem biogeochemical functions, including N or P limitation or co-limitation (Güsewell, 2004). Based on this hypothesis, Güsewell (2004) and Greenwood et al. (2008) reported that N/P ratios on a mass basis of <10 indicate limited N and ratios >20 indicate limited P, in contrast to the results of Koerselman and Meuleman (1996), who reported that the N/P ratios of <14 indicate limited N and ratios >16 indicate limited P. Yan et al. (2017) evaluated the reliability of this relationship and pointed out that the N/P ratio may indicate N (or P) limitation even when leaf N (or P) content is sufficient, whereas it may not indicate N (or P) limitation even when leaf N (or P) content is deficient. They suggested that the N/P ratios of 10 and 20 carry lower error risks than those exhibited by the ratios of 14 and 16 (Yan et al., 2017) and that such error risks should be taken into account in studies reporting N (or) P limitation based on N/P ratios. To the best of our knowledge, few studies have evaluated the N/P threshold hypothesis at a global scale (Yan et al., 2017), and there are no reports of this hypothesis considering atmospheric N and P inputs.

Assessing atmospheric N and P inputs and their relationships with plant growth under nutrient-limited or non-nutrient-limited conditions helps improve our understanding the responses of terrestrial C sequestration to frequent anthropogenic disturbances. However, it remains uncertain how drastic atmospheric pollution combined with diverse vegetation site qualities impacts plant growth (Du et al., 2020; Hou et al., 2021). For instance, in N-limited ecosystems, although the extent of the impact of N limitation remains debated, additional N deposition can alleviate plant N limitation and promotes growth (You et al., 2021). However, in non-N-limited ecosystems, excessive N deposition induces nutrient imbalances, including P deficiency, nitrate leaching, soil acidification, and the loss of base cations (Tian and Niu, 2015). Moreover, N-rich leaves are relatively more susceptible to insect and animal feeding and pathogenic infections (de Vries et al., 2014a; Gessler et al., 2017). Therefore, excessive N deposition can indirectly inhibit plant growth (de Vries et al., 2014b). Globally, P deposition has received relatively less attention than N deposition. However, since the early 20th century, industrialized countries have increasingly faced P pollution caused by the excessive use of P-based fertilizers in agriculture and intensive livestock breeding (Pan et al., 2021; Li et al., 2023). Over the past two decades, atmospheric P deposition has continued to increase in Asia and Europe (Pan et al., 2021). In addition, a few studies have suggested that P deposition may reduce the biodiversity of terrestrial ecosystems (Ceulemans et al., 2013; Fujita et al., 2014). Globally, P addition has increased plant productivity in terrestrial ecosystems by 34.9% (Hou et al., 2020). A recent study indicated that 43% of natural terrestrial ecosystems are significantly limited by P, whereas only 18% are limited by N, challenging the conventional view that N limitation is relatively more important than P limitation (Du et al., 2020). Therefore, studies focusing on P limitation may be more increasingly critical in the present scenario.

In this study, we aimed to investigate the direct and indirect mechanisms underlying the effects of atmospheric N and P deposition on the terrestrial productivity of different PFTs based on the N/P threshold hypothesis. Therefore, a dataset of 5,730 observations of different PFTs from 1970 to 2021 was established, considering a range of potential environmental drivers. An initial model based on this existing ecological knowledge was established (**Supplementary Figure S1**). We hypothesized that (1) the effects of N and P deposition on plant growth are non-linear, changing from a positive relationship with growth at low deposition to a negative relationship at high deposition through the alteration of site quality (foliar nutrient concentrations and soil pH); and (2) the growth responses (NPP) of different PFTs to atmospheric N and P deposition depend on the level of nutrient availability and are characterized by thresholds (consistent with N/P threshold hypothesis). The results obtained in this study improve our understanding of how atmospheric N and P deposition affect plant growth and thus provide valuable insights into the consequences of global environmental change and the associated impacts of acid deposition in different geographic regions.

## 2 Materials and methods

### 2.1 Stand properties

#### 2.1.1 Foliar N and P concentrations

Data for 21,620 plant species, including their leaf N and P traits, were obtained from the TRY database (https://www.try-db.org) (Kattge et al., 2011). The “life form, “ “leaf N,” and “leaf P” traits, with trait IDs 343, 14, and 15, respectively, were retrieved. The dataset collection was completed through the following steps: (1) species without corresponding coordinates were excluded; (2) species derived from greenhouses, plantations, arable land, and seedlings were removed; (3) herbs and woody plants sampled from forests and grasslands, respectively, were excluded after referring to the original papers and terrestrial ecoregion map of Olson et al. (2001) to eliminate the effects of the misjudgment of the NPP of both grasslands and forests; and (4) the Flora of China (http://frps.eflora.cn/), Tropical Plants (http://tropical.theferns.info/), Australian Native Plants (https://www.anbg.gov.au/index.html), Wikipedia (https://en.wikipedia.org/wiki), Australian National Botanic Gardens (https://www.anbg.gov.au/search/index.html), The World Flora Online (http://worldfloraonline.org/), and Useful Tropical Plants (http://tropical.theferns.info/) databases were used to identify plant functional groups and verify taxonomic classifications. To reduce the error risk in determining the nutrient limitation status caused by insufficient nutrient content in the leaves (Yan et al., 2017), data points with values deviating 2 × standard deviation (SD) were considered outliers and removed to avoid data collation or transcription errors. Subsequently, N/P ratios were calculated based on the remaining values, and data points with value deviating by 2 × SD were again removed. The final dataset contained 6,733 observations spanning the 2007–2021 duration and covering North America, Europe, Asia, and other regions affected significantly by atmospheric acid deposition. This dataset satisfied the requirements for a global-scale analysis.

#### 2.1.2 NPP

We used the global annual NPP dataset obtained from the MODIS (moderate resolution imaging spectroradiometer) NPP yearly product MOD17A3HGF v006 (available from https://earthdata.nasa.gov/). The products showed no significant bias in multiple global biomes (Turner et al., 2006) and have been extensively validated and used in global-scale research (Gang et al., 2022; Hu et al., 2022; Wang et al., 2022).

### 2.2 Climate and site quality

Climate data, including mean annual precipitation, mean annual temperature, and elevation, were extracted from WorldClim 2 (Fick and Hijmans, 2017). Soil pH data were obtained from the World Soil Database (WISE30sec: World soil property estimates at a nominal resolution of 30 by 30 arc sec) (Batjes, 2015), and the pH of the 0–20 cm surface soil was used to represent soil pH.

### 2.3 Air quality

The N deposition data were extracted from the global N deposition dataset of Ackerman et al. (2019). The P deposition data were collected through a literature search combined with extrapolation (more details are provided in note S1). All selected predictors are summarized in **Table 1**.

**Table 1:** Summary of all variables considered as predictors of net primary productivity in this study.

Group	Variable (Unit)	Abbreviation	Derived from
Response variable	Net primary productivity (g C·m^−2^·yr^−1^)	NPP	MODIS 17A3HGF (Running and Zhao, 2019); accuracy: 1 km; time range: 2007–2021
Air quality	Total N deposition (kg·ha^−1^·yr^−1^)	N dep	(Ackerman et al., 2019); accuracy: 1 km; time range: 2007–2021
	Total P deposition (kg·ha^−1^·yr^−1^)	P dep	Ensemble dataset (modeled); accuracy: 1 km; time range: 1954–2021
Climate	Mean annual precipitation (mm)	MAP	WorldClim 2 (Fick, 2017); accuracy: 1 km; time range: 2007–2021
	Mean annual temperature (°C)	MAT	WorldClim 2 (Fick, 2017); accuracy: 1 km; time range: 2007–2021
Stand property	Foliar N (mg·g^−1^)		TRY Plant Trait Database (Kattge et al., 2011); time range: 2007–2021
	Foliar P (mg·g^−1^)		TRY Plant Trait Database (Kattge et al., 2011); time range: 2007–2021
Site quality	Soil pH (pH units)		World Soil Database (WISE30sec) (Batjes, 2015); accuracy: 1 km
	Elevation (m a.s.l.)		WorldClim 2 (Fick and Hijmans, 2017); accuracy: 1 km

MODIS, moderate resolution imaging spectroradiometer; MODIS 17A3HGF, MODIS NPP data; WISE30sec, world soil property estimates at a nominal resolution of 30 by 30 arc sec.

### 2.4 Statistical analyses

Independent analytical steps were used to quantitatively and qualitatively assess the impact of global change on the NPP of terrestrial ecosystems and identify the main driving factors. All environmental predictors were unified to a 1 km spatial scale using Kriging interpolation.

#### 2.4.1 Mixed-effects model

A linear mixed-effects model (LME) was used to test the spatial changes in the NPP of different PFTs, both individually and together at a global scale. First, a univariate LME estimation of NPP was performed, with eight selected predictors as fixed effects and the vegetation site as a random effect. A quadratic term was included for each model. To reduce the variation caused by different magnitudes and to stabilize the model-fitting data, each variable was log10- transformed.

All eight predictors were included in multivariate LMEs to account for the compound effects on NPP, and the vegetation site was set as a random effect. Based on the results of the univariate LMEs and existing ecological knowledge, we included the quadratic terms of N and P deposition and their partial interactions in the multivariate LMEs. We obtained an “optimal model” using stepwise regression and the corrected Akaike information criterion (AICc), with the co-linearity of the predictors reduced by a variance inflation factor (VIF) < 0.5 (Zuur et al., 2010). To obtain the respective impacts of alternative predictors in multivariate LMEs, we extracted the partial path relationship of the selected variable, holding all other predictors at their mean. The data were *Z*-score-transformed to improve parameter estimation and ensure the comparability of regression coefficients.

#### 2.4.2 Structural equation model

The structural equation model (SEM) is widely used to verify complex ecological conjectures because of its ability to reveal direct/indirect and multiple linear relationships between variables (Grace, 2006). To explain the impact of environmental changes on NPP, we established an initial model that included the expected causal relationships between NPP and its potential predictors. The initial model was tested with four PFTs, i.e., herb (HB), evergreen broad-leaf (EB), deciduous broad-leaf (DB), and conifer (CO), both individually and together at a global scale, using leaf nutrient status (N limitation, N/P ≤10; N/P balance, 10< N/P <20; P limitation, N/P ≥20) to verify the effects of nutrient limitations on NPP. Model estimation was completed using Fisher’s criterion (*P* > 0.05, indicating that the model is acceptable) and the AICc. The random forest model (RFM) was used to rank the relative importance of the predictors. Pearson’s correlation analysis was performed to describe the impact of the predictors. In addition, the data were *Z*-score-transformed.

Data analyses were performed using R statistical software (R Core Team, 2021). We performed SEM and RFM using piecewise SEM and randomForest packages, respectively. The lmer and step functions of the lmerTest R package were used for LME construction and optimal model selection, respectively. We obtained the VIF and AICc values of the LME using the VIF function in the car R package and the AICc function in the AICcmodavg R package. We determined the univariate path relationship, holding all other variables at their mean, using the predictorEffects function in the R package.

## 3 Results

### 3.1 NPP and predictor-based variations in NPP

Based on our dataset, the mean NPP of global terrestrial ecosystems was 752 g C·m^−2^·yr^−1^, with the EB category exhibited the highest NPP at 1,016 g C·m^−2^·yr^−1^, and HB exhibited the lowest NPP at 531 g C·m^−2^·yr^−1^ (**Table 2**). Global N and P deposition were 8.18 and 0.29 kg·ha^−1^·yr^−1^, respectively, with CO being exposed to the lowest P deposition and generally lower leaf N and P contents than in the other PFTs (*P* < 0.001). In contrast, DB exhibited the highest N and P deposition and higher leaf N and P content (*P* < 0.001). The HB category generally received more N and P deposition (*P* < 0.001) and exhibited higher inherent leaf N and P contents than those in woody plants (EB, DB, and CO).

**Table 2:** Mean values ± standard deviation of net primary productivity (NPP) and growth affecting factors (air quality, climate, stand properties, and site quality).

Variable	Unit	Plant functional type			Global	
		HB	EB	DB	CO	
NPP	(g C·m^−2^·yr^−1^)	531 ± 17	1,016 ± 25	634 ± 12	773 ± 10	752 ± 14
N dep	(kg·ha^−1^·yr^−1^)	8.49 ± 0.21	7.36 ± 0.21	8.74 ± 0.26	8.51 ± 0.15	8.52 ± 0.13
P dep	(kg·ha^−1^·yr^−1^)	0.295 ± 0.004	0.279 ± 0.005	0.343 ± 0.006	0.227 ± 0.003	0.30 ± 0.003
MAP	(mm)	700 ± 20	1,667 ± 38	1,181 ± 37	773 ± 13	1,145 ± 23
MAT	(K)	280 ± 1	293 ± 0.5	291 ± 0.5	284 ± 1	288 ± 0.3
Foliar N	(mg·g^−1^)	23.7 ± 0.4	18.1 ± 0.3	20.7 ± 0.4	11.5 ± 0.2	19.32 ± 0.4
Foliar P	(mg·g^−1^)	1.94 ± 0.06	1.39 ± 0.04	1.72 ± 0.05	0.98 ± 0.03	1.56 ± 0.03
pH	(-)	6.44 ± 0.05	5.72 ± 0.03	6.02 ± 0.03	6.92 ± 0.07	6.17 ± 0.02
Elevation	(m)	1,271 ± 56	416 ± 21	368 ± 20	809 ± 34	708 ± 13
Site		194	453	251	279	631
N obs		1,908	2,224	1,598	1,003	6,733

HB, herb; EB, evergreen broad-leaf; DB, deciduous broad-leaf; CO, conifer; N dep, total N deposition; P dep, total P deposition; MAP, mean annual precipitation; MAT, mean annual temperature.

### 3.2 Non-linear relationships between NPP and N and P deposition

**Figure 1:**
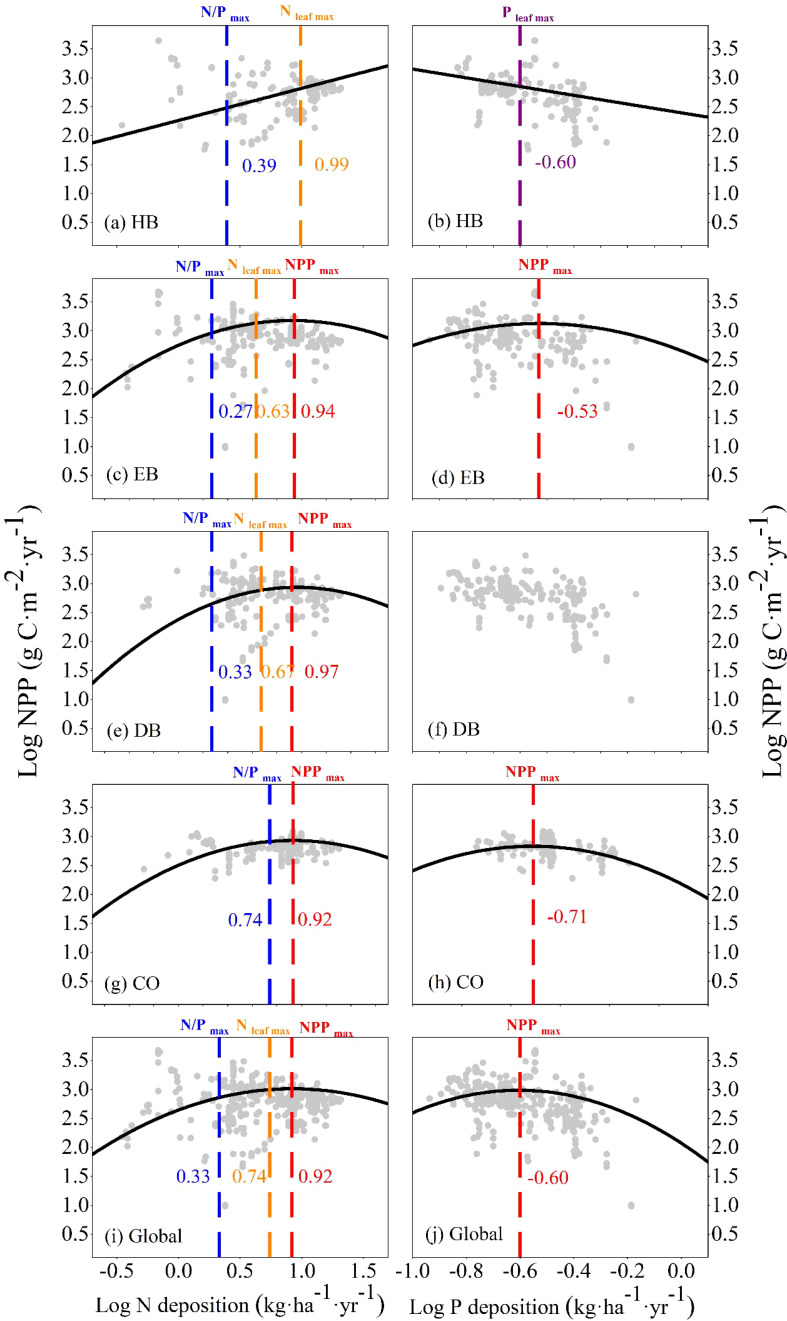
Relationship of net primary productivity (NPP) to N deposition **(A, C, E, G, I)** and P deposition **(B, D, F, H, J)** for the four plant functional types, i.e., herb, evergreen broad-leaf (EB), deciduous broad-leaf (DB), conifer (CO), both individually and at a global scale. The black line indicates the most significant fit (*P <*0.05) derived from a linear mixed-effects model per species. The red, blue, orange, and purple dotted lines/letters indicate the thresholds of the parameters NPP_max_, N/P_max_, N_leaf max_, and P_leaf max_, respectively, in the non-linear growth response.

In all PFTs, both individually and at a global scale, both univariate and multivariate LMEs confirmed that NPP was correlated non-linearly with N and P deposition, except in the HB category (**Supplementary Tables S1** and **S2**, **Table 3**, **Figure 1**). In univariate LMEs, under N deposition, the NPP thresholds of EB, DB, CO, and all four PFTs together at a global scale were 8.71, 9.33, 8.32, and 8.32 kg·ha^−1^·yr^−1^, respectively (**Figure 1**). However, a positive correlation was observed between NPP and N and P deposition in the HB (**Figure 1**). Before reaching NPP_max_, the tipping points of foliar N (N_max_) and foliar N/P ratio (N/P_max_) of EB, DB, and all four PFTs at a global scale were observed sequentially (**Figure 1**, **Supplementary Figure S2**). Under P deposition, the tipping points of the NPPs of EB, CO, and all four PFTs at a global scale were 0.30, 0.20, and 0.25 kg·ha^−1^·yr^−1^, respectively (**Figure 1**). However, only limited threshold effects of P deposition were observed on leaf nutrient contents (**Supplementary Figure S3**).

**Table 3:** Results of the optimal linear mixed-effects models with net primary productivity (NPP) as the response variable and vegetation site as a random factor for the four plant functional types, both individually and at a global scale.

Predictor	NPP				Global
	HB	EB	DB	CO	
N dep	0.04 ± 0.01^*^	0.17 ± 0.02^***^	0.07 ± 0.01^***^	0.27 ± 0.04^***^	0.23 ± 0.06^***^
N dep^2^		−0.15 ± 0.01^***^	−0.05 ± 0.01^***^	−0.13 ± 0.02^***^	−0.09 ± 0.006^***^
P dep	−0.29 ± 0.02^***^	0.58 ± 0.02^***^			
P dep^2^		−0.47 ± 0.04^***^		−0.12 ± 0.01^***^	−0.41 ± 0.02^***^
MAP	0.49 ± 0.02^***^	0.27 ± 0.02^***^		0.19 ± 0.03^***^	0.07 ± 0.02^***^
MAT	0.29 ± 0.01^***^		0.16 ± 0.02^***^	0.34 ± 0.03^***^	0.26 ± 0.01^***^
pH	−0.05 ± 0.01^***^	−0.09 ± 0.008^***^	−0.13 ± 0.01^***^	−0.15 ± 0.03^***^	−0.15 ± 0.006^***^
Foliar N	0.01 ± 0.003^***^				
Foliar P	−0.02 ± 0.003^***^		−0.008 ± 0.003^*^	0.07 ± 0.03^*^	
Elevation		−0.24 ± 0.02^***^	−0.06 ± 0.02^**^		
N dep:MAP					
N dep:pH		0.04 ± 0.01^***^		0.21 ± 0.03^***^	
N dep: Foliar N					
P dep:MAP			−0.33 ± 0.03^***^		−0.15 ± 0.02^***^
P dep: Foliar P			0.01 ± 0.004^**^		
MAT: MAP					
*R_c_^2^*	0.99	0.99	0.99	0.74	0.99
*R_m_^2^*	0.63	0.53	0.57	0.35	0.53

Displayed are the significant scaled fixed-effects estimates ± standard error and statistical significance (***P < 0.001, **P < 0.01, and *P < 0.05) of the optimal models, as well as the explained variance of the most optimal model (*R_m_^2^*, fixed effects only; *R_c_^2^*, both fixed and random effects). HB, herb; EB, evergreen broad-leaf; DB, deciduous broad-leaf; CO, conifer; N dep, total N deposition; P dep, total P deposition; MAP, mean annual precipitation; MAT, mean annual temperature.

### 3.3 Global effects of atmospheric N and P deposition on NPP

**Figure 2:**
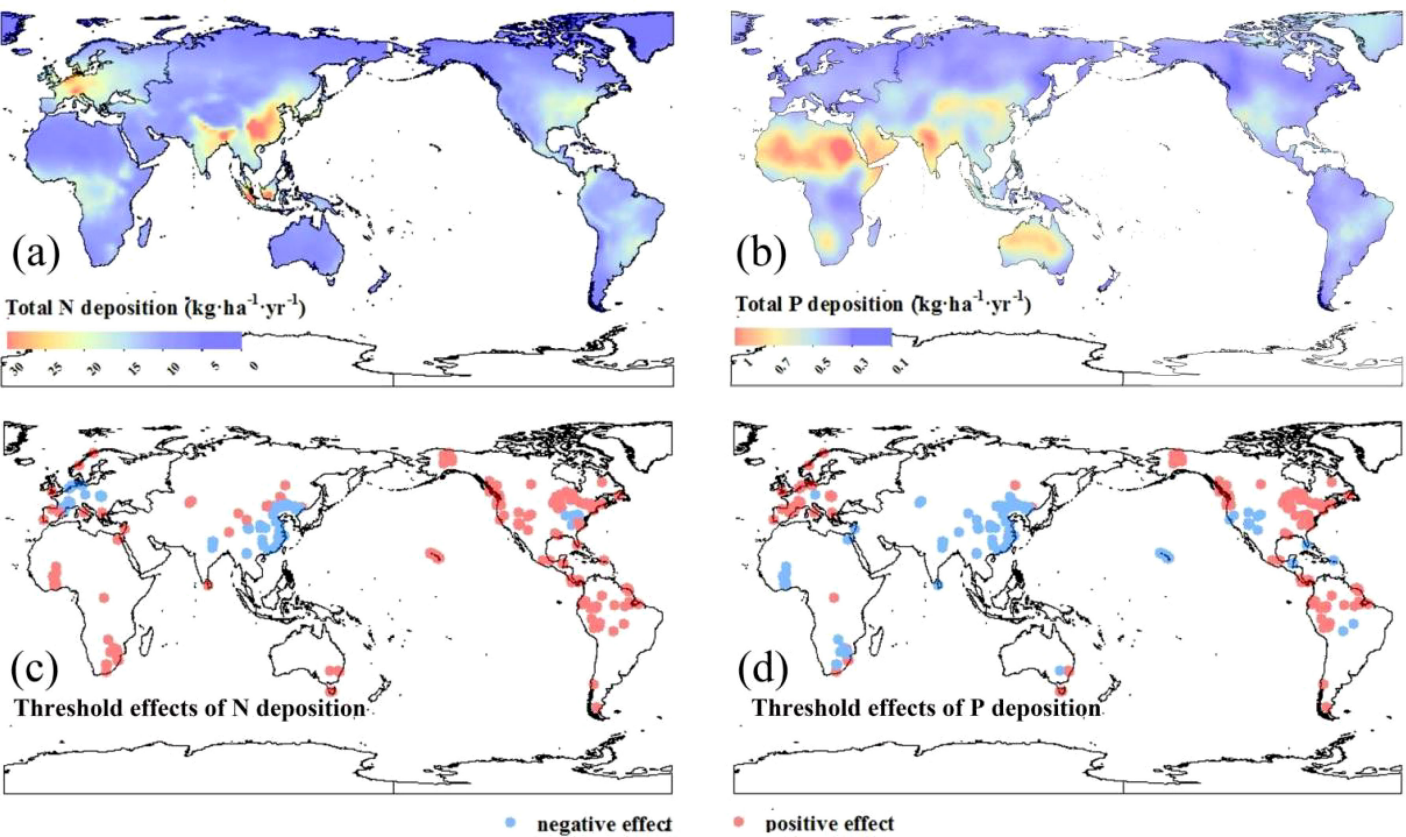
Global total N **(A)** and total P **(B)** depositions and their effects on net primary productivity are shown in **(C, D)**, respectively. Positive and negative effects were defined based on the linear mixed-effects models (see **Figures 1I, J**).

Our global maps reveal a clear pattern of N and P deposition (**Figure 2**). In this pattern, the hotspots of N deposition were mainly in eastern Asia, Europe, eastern North America, and southern Brazil (**Figure 2A**). The highest N deposition occurred in central China at 27.57 kg·ha^−1^·yr^−1^. Atmospheric P deposition is strongly associated with deserts and semiarid regions (**Figure 2B**), mainly in North Africa, India, northern Australia, and northern China. The highest P deposition was 0.96 kg·ha^−1^·yr^−1^ seen in the Sahara Desert of North Africa.

At a global scale, the impacts of N and P deposition on the NPP of terrestrial ecosystems show clear N and P limitation patterns. Globally, 53.68 and 43.88% of the terrestrial ecosystem plants were negatively affected by N and P deposition, respectively (**Figures 2C, D**). In Europe, N deposition mainly exhibited an inhibitory effect on NPP (**Figure 2C**); however, relatively low P deposition in these regions alleviated P limitation and promoted vegetation growth (**Figure 2D**). The east coast of the United States also revealed that N deposition adversely affected NPP, whereas P deposition had a positive effect (**Figures 2C, D**). It is worth noting that in China, excessive N and P deposition significantly inhibited ecosystem NPP (**Figures 2C, D**).

### 3.4 Biophysical stand properties and site quality indicators determining NPP

**Figure 3:**
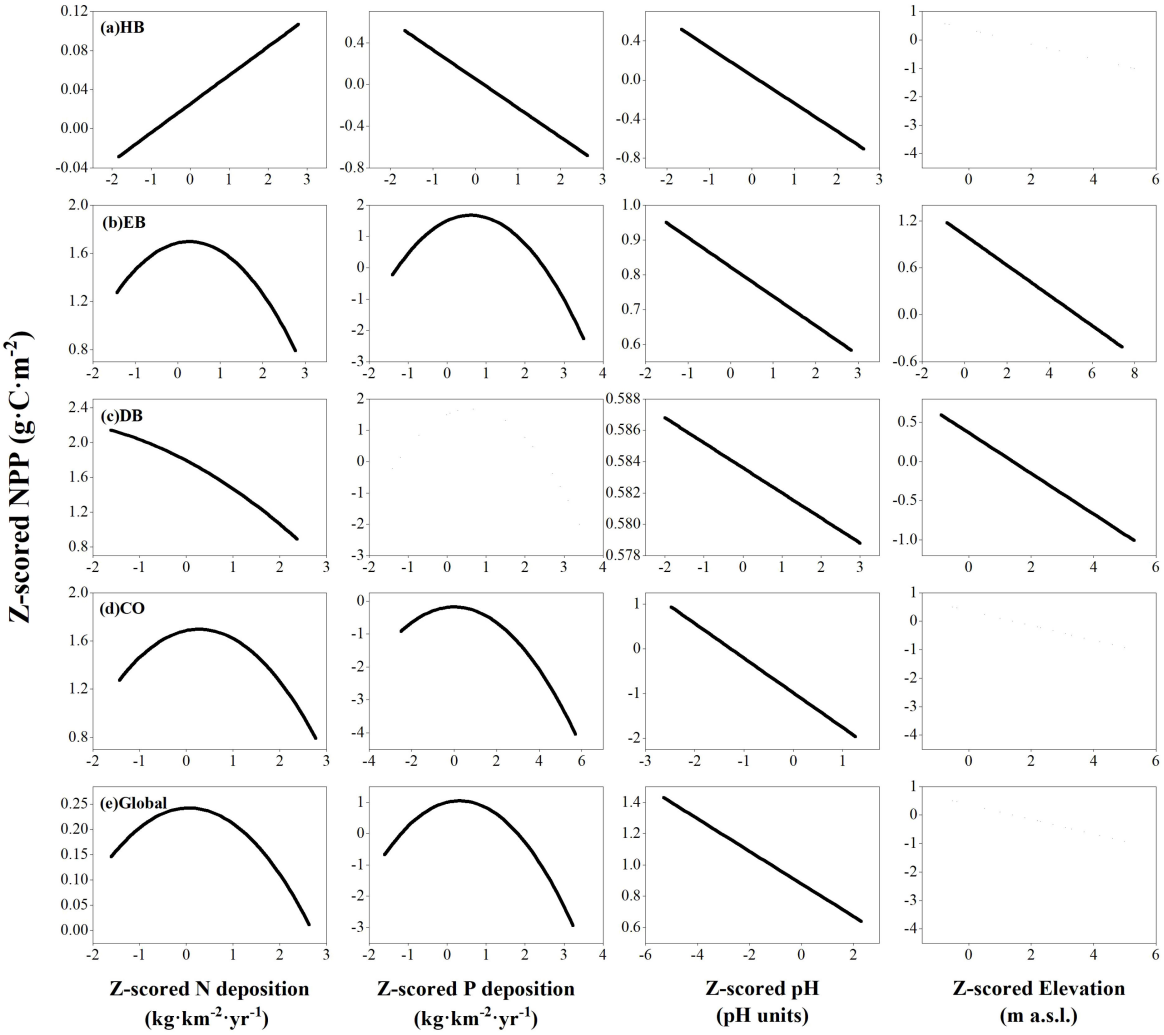
Relationships between net primary productivity (NPP) and N deposition, P deposition, soil pH, and elevation in herb **(A)**, evergreen broad-leaf **(B)**, deciduous broad-leaf **(C)**, and conifer **(D)** plant functional types (PFTs), as well as in all four PFTs together at a global scale **(E)**. Lines indicate the response of NPP to the respective variable as derived from linear mixed-effect models, holding all other predictors at their mean value, with straight lines indicating *P <*0.05.

Globally, NPP showed significant correlations with biophysical stand properties and site quality indicators in both multivariate LMEs and RFMs in all four PFTs (**Table 3**). Overall, soil pH correlated negatively with NPP (**Table 3**, **Figure 3**), through this relationship varied under nutrient-restricted conditions (**Supplementary Table S3**). Significant correlations between leaf N and P contents and NPP were observed only in HB (**Table 3**). In univariate LMEs, elevation did not exhibit a threshold effect but correlated negatively with the NPP of HB, EB, DB, and all four PFTs at a global scale (**Supplementary Table S1**). However, more specific relationships between elevation and NPP were found in multivariate LMEs, except for the EB and DB categories (**Table 3**).

### 3.5 NPP responses considering the N/P threshold hypothesis

**Figure 4:**
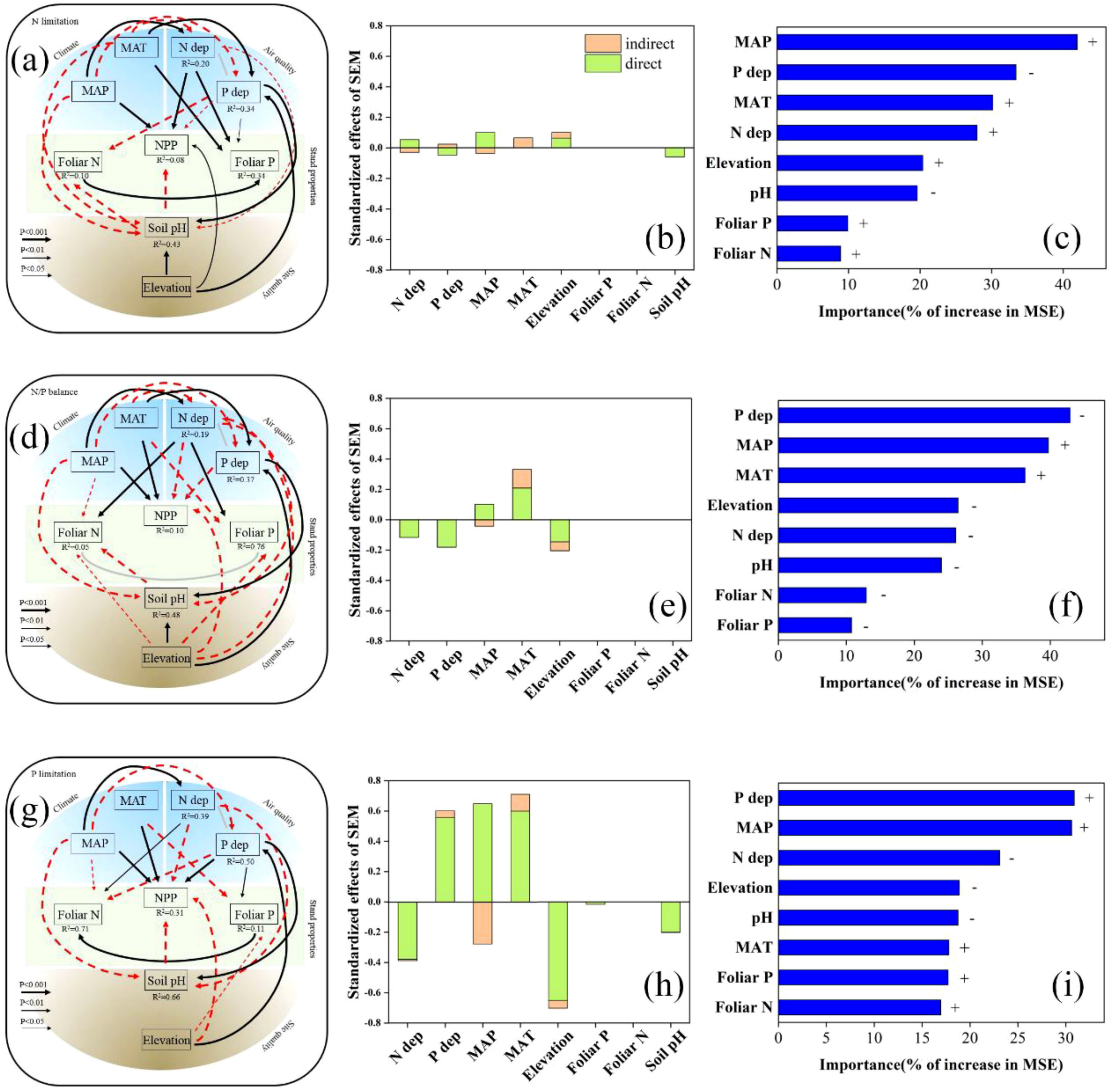
Results of the analyses using structural equation models (SEMs) and random forest models (RFMs) explaining net primary productivity (NPP) under N limitation **(A–C)**, N/P balance **(D–F)**, and P limitation **(G–I)**. Black continuous and red dashed arrows indicate significant positive and negative relationships, respectively, whereas gray lines indicate significant interactions. The width of the lines is proportional to the level of significance (*P* < 0.001, *P* < 0.01, and *P* < 0.05) (and effect size) of standardized model coefficient estimates. The coefficients of determination (*R^2^*) for the explained variables are given below the variable names. See also **Supplementary Table S3**. A greater percentage increase in mean square error (MSE) in RFMs indicates a larger relative importance. The minus signs in the results of the RFM analyses indicate a negative correlation with NPP, and the plus signs indicate a positive correlation with NPP.

The SEMs and RFMs based on the nutrient limitation hypothesis showed that PFTs under N limitation (N/P ≤10) exhibited an apparent positive response to the fertilization effects of N deposition, reflected by the positive correlation between NPP and N deposition. In this case, P deposition was negatively correlated with NPP. Under P limitation (N/P ≥20), the opposite was true for N limitation (**Figure 4**). In addition, under the N/P balance (10< N/P <20), N and P deposition correlated negatively with NPP (**Figure 4**). The RFMs revealed that P deposition was relatively more important than N deposition determining NPP under N/P balance and P limitation (**Figures 4C, F, I**). Among the PFTs, the response patterns of NPP to N and P depositions were relatively diverse. Specifically, in HB, under both N and P limitation, N and P deposition correlated positively and negatively, respectively, with NPP (**Supplementary Table S3**, **Supplementary Figure S4**); however, the results were not significant under N limitation alone (*P >*0.1). In EB, DB, CO, and all four PFTs together at a global scale, NPP exhibited similar response patterns to N and P deposition under the nutrient limitation hypothesis (**Supplementary Table S3**, **Supplementary Figures S5–S7**).

Additionally, the site quality indicators, soil pH and elevation, were strong predictors of NPP in the SEMs (**Supplementary Table S3**). Soil pH correlated negatively with NPP (*P* < 0.001). However, the elevation did not show definite patterns in the SEMs (**Supplementary Table S3**). In addition, our SEMs did not detect any direct correlations between foliar N and P contents and NPP, except in HB, where several pieces of correlated evidence were observed (**Supplementary Table S3**).

## 4 Discussion

### 4.1 Non-linear responses of NPP to atmospheric N and P deposition

Elucidating the effects of atmospheric N and P deposition on plant growth is crucial for biodiversity conservation and the maintenance of landscape heterogeneity. Numerous theoretical and empirical studies have demonstrated a “critical load” threshold in the response of plants to N deposition worldwide (Henttonen et al., 2017; Gentilesca et al., 2018; Rohner et al., 2018; Etzold et al., 2020). The most affected countries are the United States, Western Europe, Eastern Europe, South Asia, East Asia, Southeast Asia, and Japan (Dentener et al., 2006). Herein, we tested the non-linear relationships between atmospheric N and P deposition and terrestrial productivity (NPP) in the four PFTs. As expected, we found that N and P deposition non-linearly affected the NPP of woody plants globally. Studies have provided evidence for this threshold effect based on the perspectives of plant physiology and biochemistry. For example, moderate N deposition can promote plant growth; however, excess N deposition may inhibit plant photosynthesis (Li et al., 2022; Lu et al., 2023) by reducing stomatal conductance (Yao et al., 2016), chlorophyll content (Du et al., 2017), and enzyme activity (Chen et al., 2013). In addition, excess N deposition may change plant hydraulic architecture, thus affecting growth (Wang et al., 2016). Several N addition and plot control experiments (Braun et al., 2017; Salehi et al., 2021) have indicated that soil acidification and nutrient imbalances caused by excessive N deposition can inhibit plant growth. For example, in Italy and Europe, N deposition exhibited threshold effects on beech growth rates at 20 and 30 kg N·ha^−1^·yr^−1^ concentrations, respectively (Gentilesca et al., 2018; Etzold et al., 2020). A global meta-analysis based on ^15^N tracking also revealed that the N retention efficiency of forests begins to decline at 10–15 kg N·ha^−1^·yr^−1^, and N saturation is reached after exceeding 40–50 kg N·ha^−1^·yr^−1^ (de Vries et al., 2014a). Furthermore, when the N deposition in the temperate grasslands of North America (Clark and Tilman, 2008) and Inner Mongolia (Bai et al., 2010) reached 10 kg N·ha^−1^·yr^−1^ and 17 kg N·ha^−1^·yr^−1^, respectively, ecosystem productivity and biodiversity declined.

In contrast to the widespread attention paid to N deposition, the threshold effects of P deposition on vegetation growth have less frequently been reported. Recently, at the macro scale, it has been demonstrated that P limitation is not limited to tropical forests but is widely distributed globally, and its spatial pattern varies across continents, altitudes, climate zones, and aridity (Hou et al., 2020). Global-scale meta-analyses have suggested that P limitation exceeds N limitation to become the primary nutrient limitation in natural ecosystems (Du et al., 2020). At the microscopic scale, studies have shown that moderate P addition can significantly change the soil arbuscular mycorrhizal fungal community composition, leading to a decrease in mycorrhizal diversity (Camenzind et al., 2014; Duenas et al., 2020) and alleviate the inhibition of N deposition on photosynthesis (Hao et al., 2023). In this study, we demonstrated that P deposition also exhibited a threshold effect on plant growth. Our study further suggest an increasing P limitation in global terrestrial ecosystems, likely because of a relatively high atmospheric N/P deposition ratio, which enhances P limitation in the ecosystem (Duenas et al., 2020). Nevertheless, our results suggest that accounting for N and P deposition in a multiscale theoretical framework can provide a sound basis for sustaining terrestrial C sequestration. However, due to the small volume of atmospheric P deposition before 2000 (no more than 1.5 kg P·ha^−1^·yr^−1^, Pan et al., 2021), the tipping point of NPP caused by P deposition in this study may be smaller, but the observed threshold effect is real. In future studies, it is necessary to collect more samples of P deposition for model fitting.

The sequential threshold effects of leaf N/P ratio, leaf N and P contents, and NPP on N deposition at the leaf and individual plant scales indicate that the effects of N deposition on plant growth may be gradual and accumulated and that leaf nutrient status may be associated with plant growth. However, our results revealed that leaf N and P concentrations were significantly correlated with the NPP of HB alone (**Supplementary Table S3**). This finding can be attributed to the rapid growth and metabolic rate in HB, maintained by several ribosomes and intensive protein synthesis, leading to an accumulation of rRNA in the cell and correspondingly higher P content and lower N/P ratio (Harris, 2003). Therefore, HB is more likely to show higher demand for N and excess P under atmospheric N and P fertilization. Our results support the productivity–nutrient allocation hypothesis, which states that herbs allocate relatively more nutrients for growth, whereas woody plants additionally allocate nutrients for morphological development (Tang et al., 2018); thus, the leaf nutrient concentrations of HB were more closely related to NPP.

### 4.2 N/P threshold hypothesis applies to the growth of woody plants but not to that of herbs

N and P are the major limiting nutrients in ecosystems worldwide; thus, ecosystems can be generally classified as N- or P-limited or N/P-balanced ecosystems (Li et al., 2016; Zhu et al., 2022). For instance, N addition can significantly increase foliar N concentrations, and subsequently, P in plant leaves is assimilated to promote plant growth under N-limited conditions (Cleveland et al., 2013). However, under P-limited conditions, plants increase their P demand and uptake to maintain the stability of their internal chemical stoichiometry when foliar N concentrations are excessively increased by N addition (Marklein and Houlton, 2012). In addition, PFTs can regulate the stoichiometry of foliar N and P in response to N addition because of the different biological and ecological characteristics required for adapting to environmental changes (Townsend and Asner, 2013; Tian et al., 2016). For instance, herbaceous plants exhibit lower nutrient use efficiency but higher plant growth rates and plasticity, with relatively more sensitivity to N addition than observed for woody plants (Gilliam et al., 2016; Tian et al., 2016). A global meta-analysis based on the N/P threshold hypothesis indicated that increasing N deposition may aggravate plant P limitation under N-limited conditions but improve P limitation under P-limited conditions, and the effects are relatively more pronounced in herbaceous plants (You et al., 2018). Testing the N/P threshold hypothesis, we found that N and P deposition promoted the growth of woody plants under N and P limitation, respectively (**Supplementary Table S3**), suggesting that land management decisions to control the N/P deposition ratio are critical for alleviating plant nutrient constraints and maintaining ecosystem productivity.

### 4.3 Effects of site quality on NPP

We found that soil pH correlated negatively with NPP at a global scale but varied considerably under nutrient restriction conditions (**Supplementary Table S3**). This negative correlation between soil pH and NPP can be attributed to soil acidification, reducing species diversity and abundance but increasing N availability for plants globally (Gilliam, 2006; Du et al., 2014; Midolo et al., 2019). In general, soil acidification is regulated by different acid buffer compounds, including carbonate at pH >7, soil exchangeable base cations (EBCs) at 4.5< pH <7, and aluminum compounds at pH <4.5 (Bowman et al., 2008; Lieb et al., 2011). In this study, global soil pH values ranged from 5.72 to 6.92, indicating that EBCs are the main acid buffer compounds in the soil, and no apparent aluminum toxicity was observed. Therefore, a decline in soil pH and a rise in available soil N content promote plant growth. PFT-based variations may result from differences at finer spatial scales. For example, tropical forests generally exhibit the highest NPP but lower soil pH (acidic soil) than recorded for other forest types because litter in tropical forests is not converted into soil organic matter, with its decomposition completed by fungi and other benthic organisms. Long-term N deposition tends to reduce cation saturation in tropical forest soils, reducing their acid-neutralizing capacity, and does not cause aluminum toxicity (Lu et al., 2014). Our results indicate that soil acidification promotes tree growth globally and that it may be relatively more appropriate to incorporate actual microenvironments (i.e., N- or P-limited) at the regional scale.

Temperature limitations (low temperatures) associated with increases in elevation can directly inhibit plant photosynthesis (Li et al., 2008) and meristem activity (Streit et al., 2013) or indirectly impact plant growth through soil nutrient availability (e.g., soil mineralization correlates positively with soil temperature) (Carbutt et al., 2013). Distinguishing the direct and indirect effects of elevation on plant growth remains challenging. A few studies have shown that an increase in elevation results in higher foliar N concentrations (Peng et al., 2012) but lower P concentrations (Gerdol et al., 2017), which is consistent with the results reported in this study (**Supplementary Figure S8**). These results support the biogeochemical hypothesis, which states that low temperatures at high elevations inhibit nutrient extraction in roots and reduce N availability by reducing organic decomposition and mineralization (Güsewell, 2004; Reich and Oleksyn, 2004). In this study, univariate LMEs showed that NPP exhibited negative responses with increasing elevation (**Supplementary Table S1**); however, these relationships disappeared after considering additional predictors in multivariate LMEs, likely because when studies are conducted at relatively large scales, variations in the distribution patterns of water and heat (e.g., latitude patterns) may be more important than elevation.

In addition, PFTs may be another key factor in determining the effects of elevation on plant nutrient contents (Zhao et al., 2018; Kulkarni et al., 2023). For example, Korner et al. (1986) reported that leaf N concentrations in herbaceous plants, but not in evergreen woody plants, increase with increasing altitudinal elevation in New Zealand. In contrast, Soethe et al. (2008) reported that the N, P, K, and S contents in shrubs and herbs decrease significantly with increasing altitudinal elevation. These diverse results have been attributed to differences in the adaptation strategies of different PFTs in response to elevational changes because of differences in community clusters and altitudinal environments between studies (Munson et al., 2022; Zhang et al., 2022; Jaroszynska et al., 2023). However, the results obtained in our study indicate that leaf N and P contents in different PFTs respond weakly to changes in elevation (**Supplementary Figures S4–S7**). An integrated study of other indirect effects of elevation on the growth of PFTs may provide additional observational perspectives not readily obtained in species-specific studies.

## 5 Conclusions

We confirmed the threshold effects of atmospheric N and P deposition on C sequestration in woody plants based on an extensive dataset of different PFTs distributed worldwide. In addition to the driving forces exerted by site quality, N and P deposition may be as important as climate in affecting NPP at a global scale. The results of our study also proved that the N/P threshold hypothesis is feasible in determining how atmospheric N and P deposition affect the growth of woody plants through nutrient limitation. Based on these results, we suggest that policies for controlling atmospheric deposition must be based on the characteristics of different PFTs or biomes, as their “critical load” thresholds to the atmospheric environment differ.

## Data Availability

The original contributions presented in the study are included in the article/**Supplementary Material**. Further inquiries can be directed to the corresponding authors.
