# Supplementary material for: A global analysis of plant nutrient limitation affected by atmospheric nitrogen and phosphorous deposition
Source: Front Plant Sci. 2024 Dec 19;15:1473493. doi: 10.3389/fpls.2024.1473493 (PMC11693671; doi:10.3389/fpls.2024.1473493)
Supplement: Supplementary file 1 [file DataSheet1.docx]

SUPPLEMENTARY INFORMATION

**A global analysis of plant nutrient limitation affected by atmospheric nitrogen and phosphorous deposition**


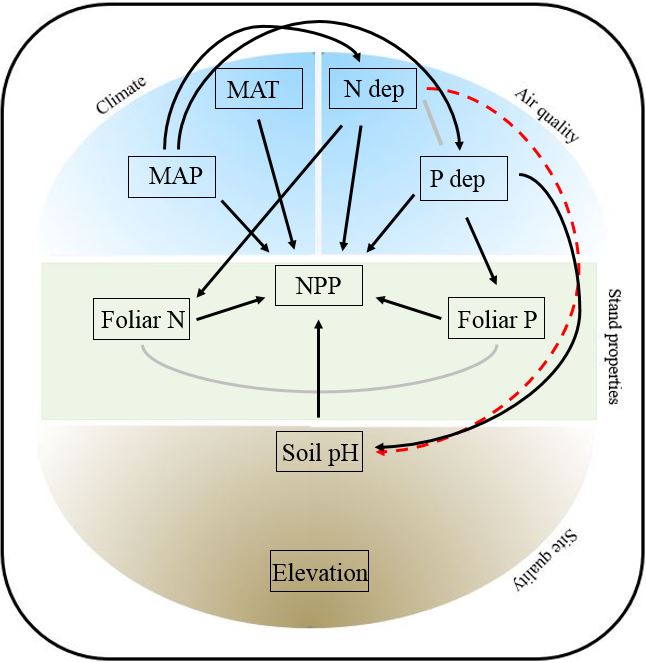


**Figure S1** Initial SEM model. NPP: net primary productivity; MAP: precipitation; MAT: temperature; Foliar N: foliar N concentration; Foliar P: foliar P concentration; Soil pH: pH of mineral soil horizons; N dep: N deposition; P dep: P deposition. Expected positive relationships are indicated by continuous black arrows; and negative relationships by dashed red arrows; interaction only indicated by gray lines.


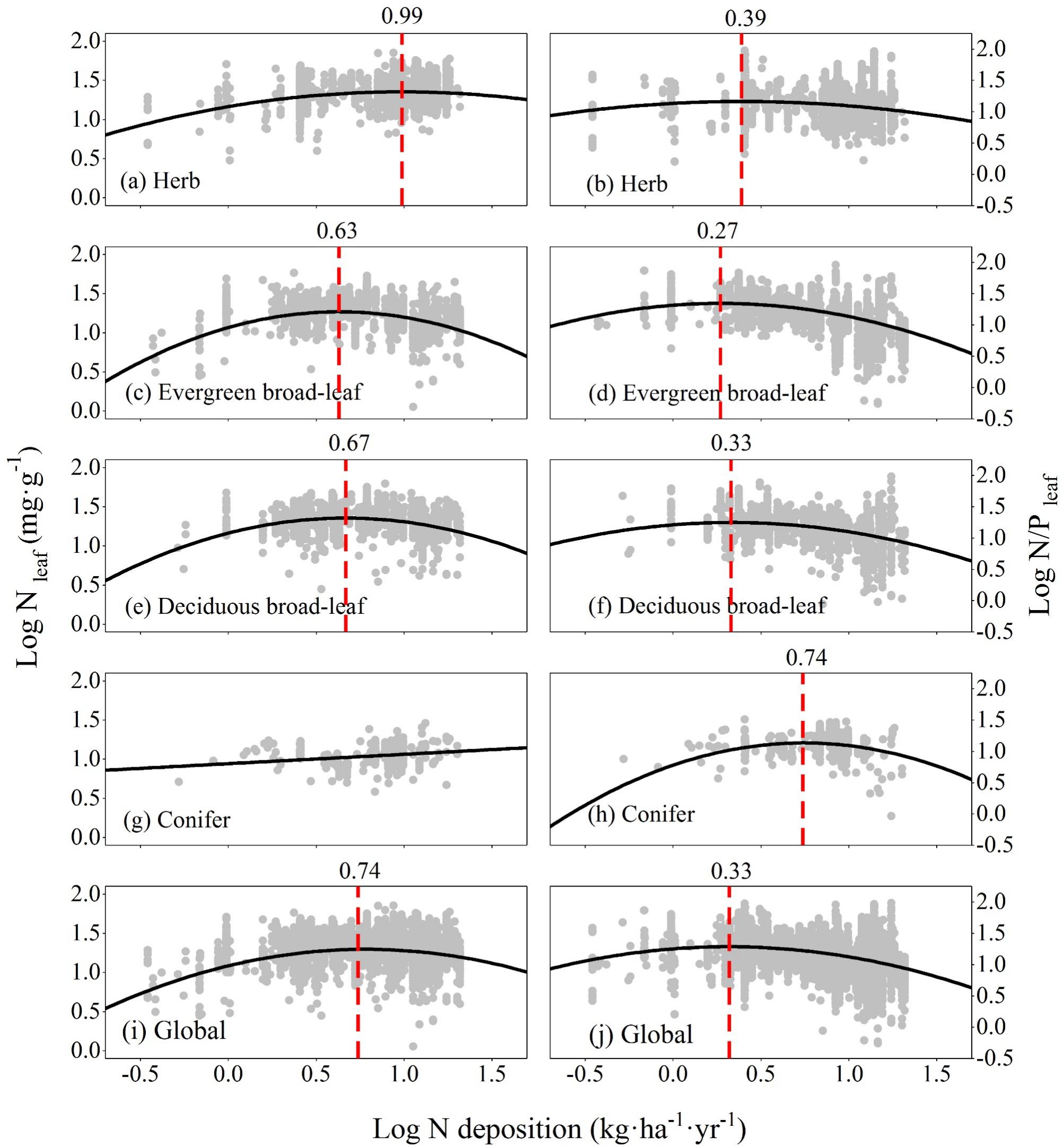


**Figure S2** Relationship of leaf N content (a, c, e, g, i) and leaf N/P ratio (b, d, f, h, j) to N deposition for different functional groups (Herb, EB, DB, CO, and Global scale).The black solid line indicates the best significant fit (*P* < 0.05) derived from linear mixed effects model (LME) per species. The red dash line indicates the threshold of non-linear growth response.


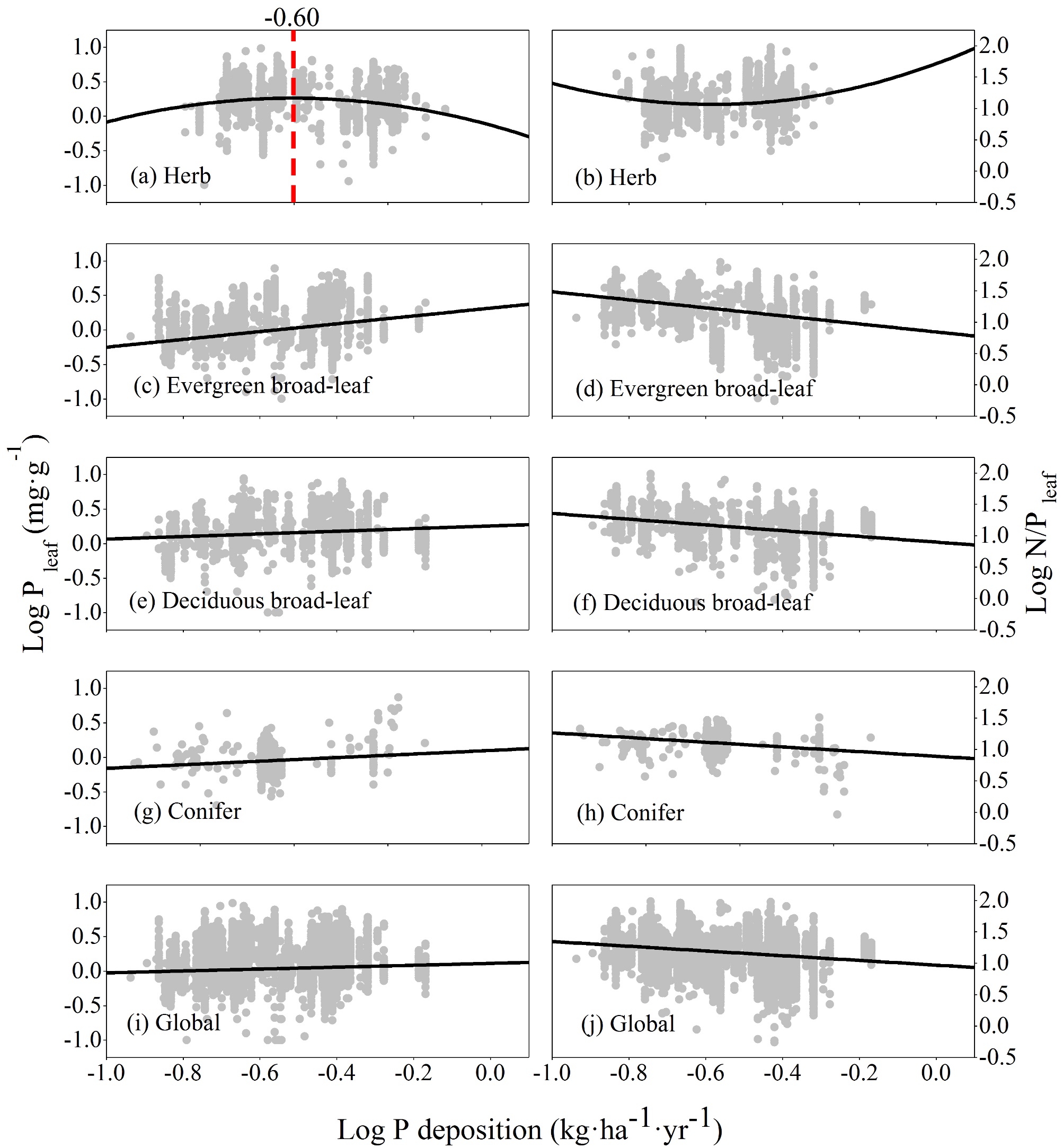


**Figure S3** Relationship of leaf P content (a, c, e, g, i) and leaf N/P ratio (b, d, f, h, j) to P deposition for different functional groups (Herb, EB, DB, CO, and Global scale).The black solid line indicates the best significant fit (*P* < 0.05) derived from linear mixed effects model (LME) per species. The red dash line indicates the threshold of non-linear growth response.


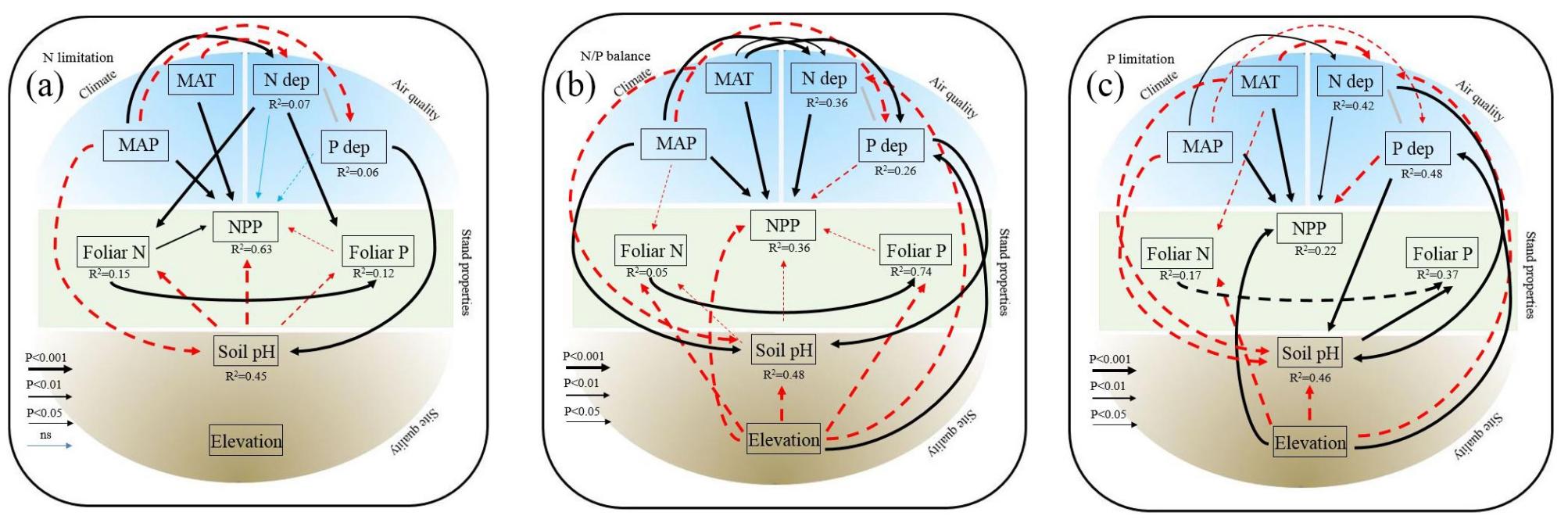


**Figure S4** Structural equation models (SEM) of Herb to explain NPP under N limitation (a), N/P balance (b), and P limitation (c). Significant positive relationships are indicated by black continuous arrows, negative relationships indicated by red dashed arrows, and interaction only indicated by gray lines. Some ns (not significant relationships) results indicated by blue arrow. Width of the lines is proportional to the significance (****P* < 0.001, ***P* < 0.01, **P* < 0.05) (and effect size) of standardized model coefficient estimates. Coefficients of determination (R^2^) for explained variables are given below the variable names.


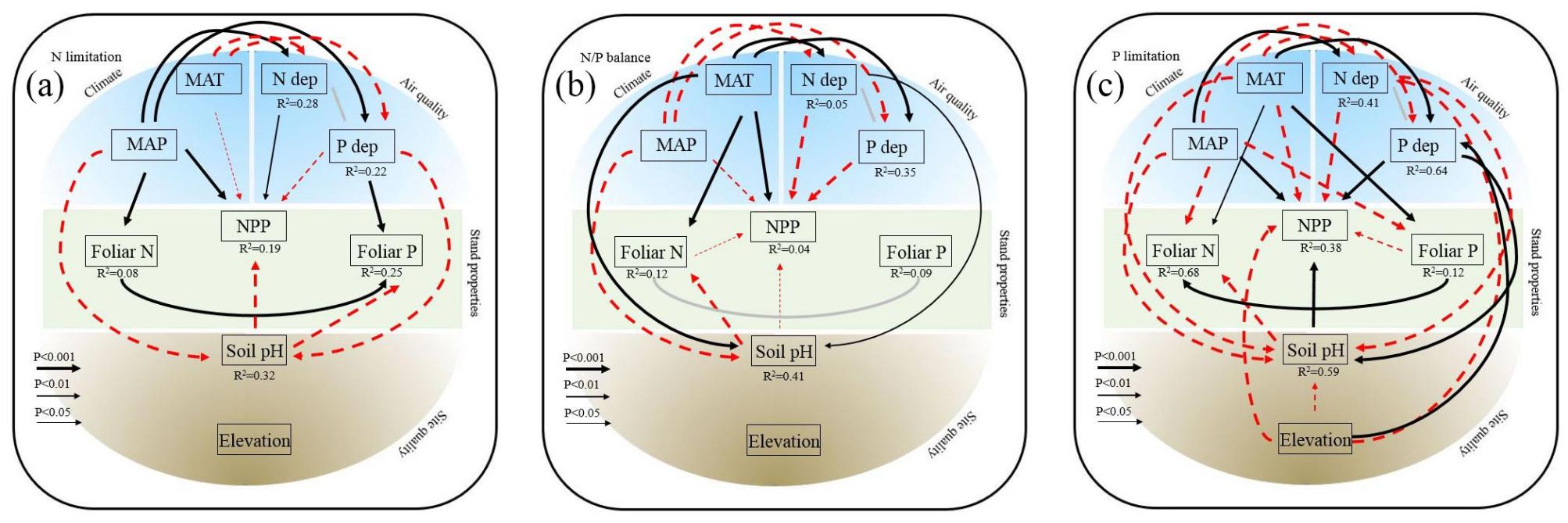


**Figure S5** Structural equation models (SEM) of evergreen broad-leave to explain NPP under N limitation (a), N/P balance (b), and P limitation (c). Significant positive relationships are indicated by black continuous arrows, negative relationships indicated by red dashed arrows, and interaction only indicated by gray lines. Width of the lines is proportional to the significance (****P* < 0.001, ***P* < 0.01, **P* < 0.05) (and effect size) of standardized model coefficient estimates. Coefficients of determination (R^2^) for explained variables are given below the variable names.


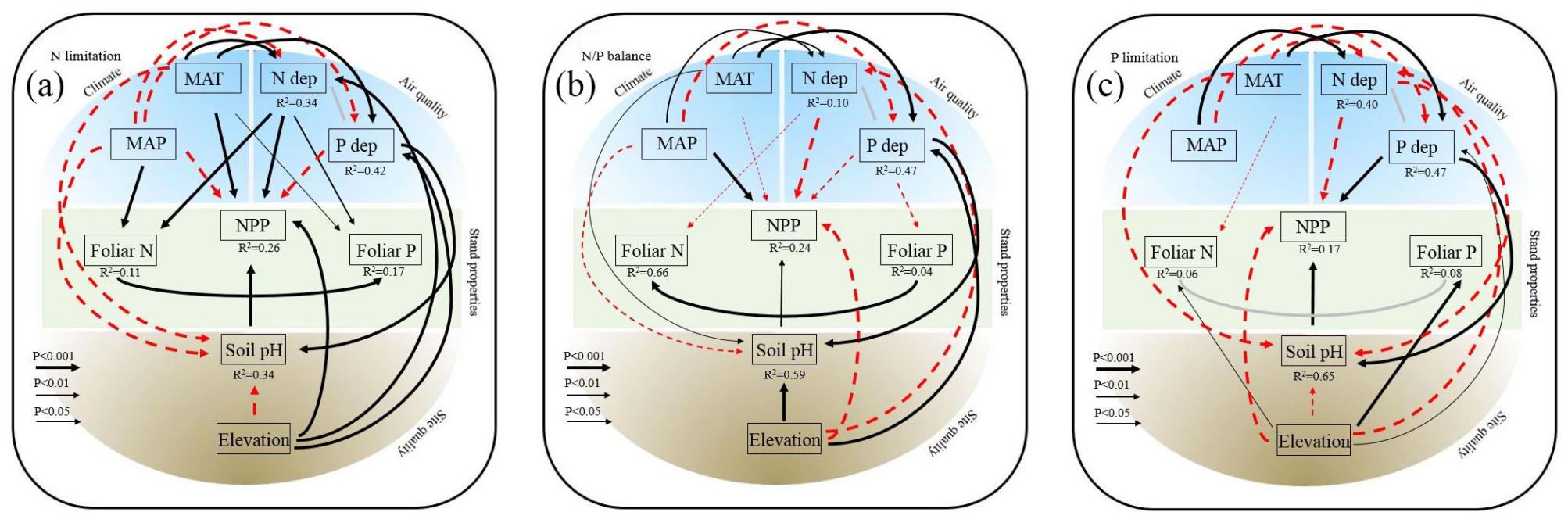


**Figure S6** Structural equation models (SEM) of deciduous broad-leave to explain NPP under N limitation (a), N/P balance (b), and P limitation (c). Significant positive relationships are indicated by black continuous arrows, negative relationships indicated by red dashed arrows, and interaction only indicated by gray lines. Width of the lines is proportional to the significance (****P* < 0.001, ***P* < 0.01, **P* < 0.05) (and effect size) of standardized model coefficient estimates. Coefficients of determination (R^2^) for explained variables are given below the variable names.


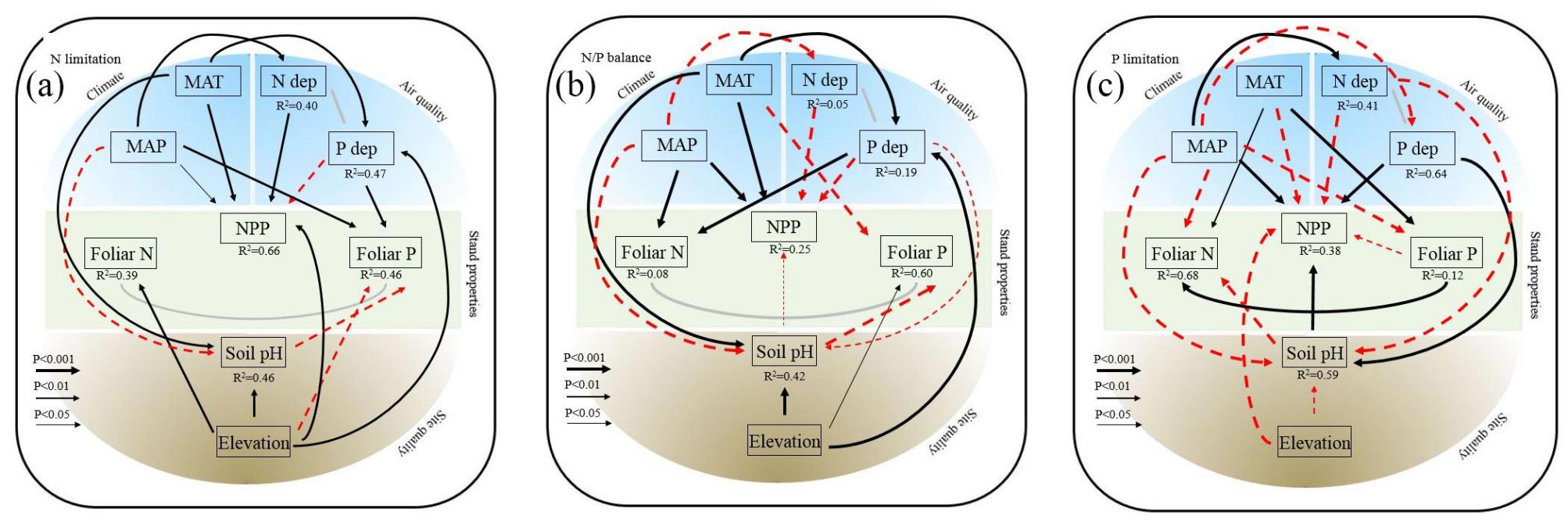


**Figure S7** Structural equation models (SEM) of conifer to explain NPP under N limitation (a), N/P balance (b), and P limitation (c). Significant positive relationships are indicated by black continuous arrows, negative relationships indicated by red dashed arrows, and interaction only indicated by gray lines. Width of the lines is proportional to the significance (****P* < 0.001, ***P* < 0.01, **P* < 0.05) (and effect size) of standardized model coefficient estimates. Coefficients of determination (R^2^) for explained variables are given below the variable names.


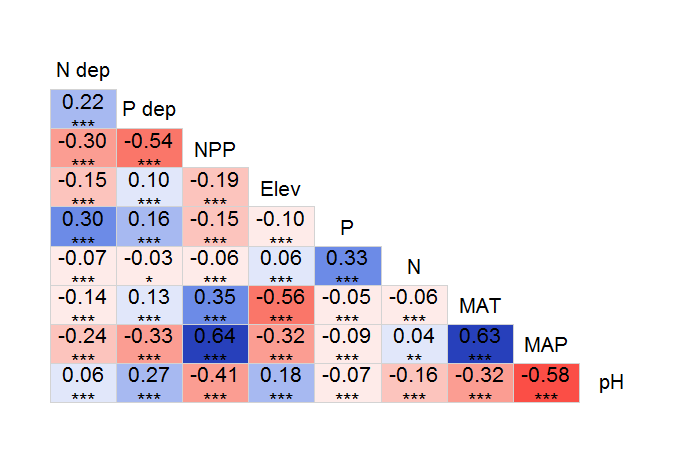


**Figure S8** Pearson correlations between 8 selected indicators and NPP across global scale. N dep, N deposition; P dep, P deposition; NPP, Net primary productivity; Elev, elevation; P, foliar P; N, foliar N; MAT, mean annual temperature; MAP, mean annual precipitation; pH, soil pH. Significance were indicated by ****P* < 0.001, ***P* < 0.01, **P* < 0.05.

**Table S1** Significant coefficients (x^2^, x) of mixed effects models with NPP as response variable and site as random factor. Significant level were indicated by ****P* < 0.001, ***P* < 0.01, and **P* < 0.05. Model selection between liner and non-liner mixed effect model were summarized in Table S2.

| Predictor (x^2^, x) | NPP | | | | |
| --- | --- | --- | --- | --- | --- |
|  | Herb | EB | DB | CO | Global |
| N dep | 0.55x^***^ | -0.49x^2***^+0.92x^***^ | -0.60x^2***^+1.16x^***^ | -0.48x^2***^+0.88x^*^ | -0.43x^2***^+0.79x^***^ |
| P dep | -0.76x^*^ | -1.69x^2***^-1.78x^***^ | n.s. | -1.79x^2***^-2.55x^***^ | -2.50x^2***^-3.03x^***^ |
| MAP | 0.37x^***^ | 0.05x^***^ | 0.02x^***^ | 0.11x^***^ | 0.02x^***^ |
| MAT | 8.43x^*^ | 5.46x^*^ | 1.84x^*^ | 0.96x^*^ | 1.95x^*^ |
| pH | -2.31x^2***^+1.9x^**^ | -1.38x^2*^+1.28x. | -2.24x^2***^+3.46x^***^ | -0.09x^**^ | 1.62x^2***^-3.27x^***^ |
| Foliar N | n.s. | -0.02x^*^ | -0.01x^*^ | n.s. | 0.01x^*^ |
| Foliar P | 0.07x^2***^+0.02x^*^ | 0.05x^2***^ | n.s. | 0.04x^**^ | 0.07x^2***^ |
| Elevation | -0.12x^***^ | -0.11x^***^ | -0.07x^***^ | n.s. | -0.1x^***^ |

**Table S2** Response of NPP to selected predictors based on comparison of linear and non-linear mixed-effects models.

| **Predictors** | **Herb** | | | **EB** | | | **DB** | | | **CO** | | | **Global** | | |
| --- | --- | --- | --- | --- | --- | --- | --- | --- | --- | --- | --- | --- | --- | --- | --- |
|  | R_m_^2^_liner_/ R_m_^2^_non-liner_ | ANOVA | | R_m_^2^_liner_/ R_m_^2^_non-liner_ | ANOVA | | R_m_^2^_liner_/ R_m_^2^_non-liner_ | ANOVA | | R_m_^2^_liner_/ R_m_^2^_non-liner_ | ANOVA | | R_m_^2^_liner_/ R_m_^2^_non-liner_ | ANOVA | |
|  |  | AIC_liner_/ AIC_non-liner_ | *P* |  | AIC_liner_/ AIC_non-liner_ | *P* |  | AIC_liner_/ AIC_non-liner_ | *P* |  | AIC_liner_/ AIC_non-liner_ | *P* |  | AIC_liner_/ AIC_non-liner_ | *P* |
| **N dep** | 0.22/0.24 | -4963/-4964 | ** | 0.29/0.36 | -5220/-5297 | *** | 0.22/0.37 | -4904/-5122 | *** | 0.29/0.33 | -2426/-2528 | *** | 0.31/0.36 | -9226/-9292 | *** |
| **P dep** | 0.14/0.13 | -4580/-4576 | ****** | 0.31/0.42 | -5179/-5195 | *** | n.s./ n.s. | - | - | 0.16/0.77 | -2521/-2543 | *** | 0.23/0.48 | -9235/-9259 | *** |
| **MAP** | 0.43/0.13 | -5042/-4520 | *** | 0.13/0.07 | -5744/-5210 | *** | 0.36/0.12 | -4832/-4726 | *** | 0.19/0.09 | -2608/-2530 | *** | 0.46/0.15 | -9238/-9194 | *** |
| **MAT** | 0.18/0.06 | -4847/-4402 | *** | 0.21/0.18 | -5364/-5357 | ** | 0.51/0.36 | -4979/-4766 | *** | 0.54/0.21 | -2558/-2514 | *** | 0.35/0.24 | -9369/-9205 | *** |
| **pH** | 0.04/0.05 | -4675/-4669 | *** | 0.07/0.08 | -5415/-5411 | * | 0.05/0.07 | -4739/-4726 | *** | 0.01/0.09 | -2519/-2417 | ** | 0.-6/0.18 | -9246/-9245 | * |
| **Foliar N** | n.s./ n.s. | - | - | 0.09/0.07 | -5183/-5179 | * | 0.08/0.06 | -4735/-4729 | * | n.s./ n.s. | - | - | 0.12/0.06 | -9927/-9913 | ** |
| **Foliar P** | 0.09/0.12 | -4159/-4169 | *** | 0.08/0.13 | -5185/-5199 | *** | n.s./ n.s. | - | - | 0.30/0.17 | -2572/-2423 | *** | 0.08/0.15 | -9967/-9932 | *** |
| **Elevation** | 0.12/0.07 | -4459/-4697 | *** | 0.42/0.18 | -5550/-5530 | *** | 0.27/0.10 | -5039/-4866 | *** | n.s./ n.s. | - | - | 0.39/0.21 | -9298/-9244 | *** |

Since the presence of R_c_^2^ > 0.95 in most regressions, we only report R_m_^2^ here for model comparison. R_m_^2^_liner_ and R_m_^2^_non-liner_ represent fixed effects only of liner and non-liner mixed effect model, respectively; AIC_liner_ and AIC_non-liner_ represent the Akaike Information criterion (AIC) of liner and non-liner fixed effect models, respectively. The significance of model comparison was indicated by ****P* < 0.001, ***P* < 0.01, **P* < 0.05.

**Table S3** Performance of SEMs for PFTs and coefficients of variables (effect size) with significant (****P* < 0.001, ***P* < 0.01, **P* < 0.05, .*P* < 0.1) impact on NPP.

|  | PFT | | | | | | | | | | | | Global | | |
| --- | --- | --- | --- | --- | --- | --- | --- | --- | --- | --- | --- | --- | --- | --- | --- |
|  | Herb | | | EB | | | DB | | | CO | | |  |  |  |
|  | N-limitation | N/P-balance | P-limitation | N-limitation | N/P-balance | P-limitation | N-limitation | N/P-balance | P-limitation | N-limitation | N/P-balance | P-limitation | N-limitation | N/P-balance | P-limitation |
| Fisher’s C | 23.66 | 17.856 | 20.607 | 28.23 | 21.317 | 26.277 | 24.368 | 23.884 | 31.778 | 43.506 | 27.851 | 25.732 | 26.852 | 11.868 | 12.094 |
| P | 0.166 | 0.597 | 0.762 | 0.168 | 0.094 | 0.05 | 0.328 | 0.583 | 0.284 | 0.084 | 0.181 | 0.132 | 0.217 | 0.854 | 0.599 |
| dF | 18 | 20 | 26 | 22 | 14 | 16 | 22 | 26 | 28 | 32 | 22 | 27 | 22 | 18 | 14 |
| N | 604 | 861 | 443 | 512 | 859 | 853 | 420 | 806 | 372 | 240 | 408 | 355 | 1536 | 2526 | 1668 |
| AIC | 91.66 | 97.86 | 94.61 | 94.23 | 93.32 | 110.28 | 102.37 | 97.88 | 101.78 | 115.51 | 105.85 | 112.76 | 104.85 | 93.87 | 98.09 |
| **Variables with significant impact on NPP** | | | | | | | | | | |  |  |  | | |
| N-deposition | 0.06. | -0.10^***^ | 0.36^**^ | 0.29^**^ | -0.13^***^ | -0.23^***^ | 1.07^***^ | -0.16^***^ | -0.18^***^ | 0.28^***^ | -0.26^***^ | -0.21^***^ | 0.05^***^ | -0.12^***^ | -0.38^***^ |
| P-deposition | -0.01. | -0.10^***^ | -0.29^**^ | -0.29^**^ | -0.22^***^ | 0.79^***^ | -1.38^***^ | -0.18^**^ | 0.31^***^ | -0.55^***^ | -0.28^***^ | 0.75^***^ | -0.05^**^ | -0.18^***^ | 0.56^***^ |
| MAP | 0.54^***^ | 0.36^**^ | 0.43^***^ | 0.61^***^ | -0.14^**^ | 1.07^***^ | -0.08^***^ | 0.57^***^ |  | 0.08^*^ | 0.19^***^ | 1.03^***^ | 0.10^***^ | 0.10^***^ | 0.65^***^ |
| MAT | 0.16^***^ | 0.08^***^ | 0.33^***^ | -0.17^***^ | 0.27^***^ | -0.41^***^ | 0.64^***^ | -0.13^*^ |  | 0.49^***^ | 0.34^***^ | -0.31^***^ |  | 0.21^***^ | 0.59^***^ |
| Elevation |  | -0.26^***^ | 0.55^***^ |  |  | -0.38^***^ | 0.79^***^ | -0.12^***^ | -0.22^***^ | 0.19^***^ |  | -0.35^***^ | 0.06^**^ | -0.15^***^ | -0.65^***^ |
| Foliar P | -0.01^*^ | -0.01^*^ |  |  |  | -0.02^**^ |  |  |  |  |  | -0.02^**^ |  |  | 0.02^***^ |
| Foliar N | 0.02^**^ |  |  |  | -0.02^*^ |  |  |  |  |  |  |  |  |  |  |
| pH | -0.14^***^ | -0.06^*^ |  | -0.08^***^ | -0.02^*^ | 0.09^***^ | 0.02^***^ | 0.04^**^ | 0.16^***^ |  | -0.08^*^ | 0.07^***^ | -0.06^***^ |  | -0.20^***^ |

**Note S1** **Robust regression was employed to fit P deposition**

The existing dataset of Tipping *et al.* (2014) covers the period from 1954 to 2012 and includes 253 observations of total P deposition (TP), filtered total P deposition (FTP), and inorganic P (PO_4_-P). Tipping *et al.* (2014) estimated a global P budget of 3.7 Tg yr^−1^, with a P deposition to land of 0.27 kg ha^−1^ yr^−1^. With a chemical transport model, however, Mahowald *et al.* (2008) simulated a global atmospheric P deposition of only 1.39 Tg yr^−1^. This value is less than 50% of the global deposition pool interpolated from measurements, highlighting a poor agreement between modeled and observed atmospheric P deposition. While the gap can be filled by an increase in combustion-related emissions in the model (Wang *et al.*, 2015b, Wang *et al.*, 2017b), uncertainties still exist in the previous observational compilations. As Tipping *et al.* (2014) recognized, although their data covered most land surfaces of the globe, they were biased towards sites in North America and Europe (n = 209, or 83% of the measurement locations). For Asia, however, their database only compiled eight locations, which was a large omission of observations, especially in China. Therefore, a literature search was conducted, and 125 samples from 1962 to 2021 were obtained as supplementary. In particular, we included data compilations for the period 1979 to 2021 in China (n = 51). All data were converted to TP with reference to PO_4_-P: TP≈1:1 and FTP: TP≈19:27 from Tipping *et al.* (2014), and wet P deposition: dry P deposition≈1:1 from Pan *et al.* (2021). Finally, global P deposition was obtained via extrapolation

To obtain the global pattern of atmospheric P deposition, we selected climate variables (MAT, MAP, precipitation seasonality (Ps), temperature seasonality (Ts), precipitation in wet season (Pwet), temperature in wet season (Twet)), soil fertility (N deposition, soil total P (Soil P), soil clay (Clay), soil pH, cation exchange capacity (CEC), aluminum saturation (ALSA), and NPP as predictors. Ps, Ts, Pwet, and Twet were obtained from WorldClim 2 (Fick & Hijmans, 2017). Soil clay, pH, CEC, and ALSA were obtained from World Soil Database (WISE30sec) (Batjes, 2015). Soil P was obtained from an online world soil data set (Shangguan, 2014; available at http://globalchange.bnu.edu.cn.).

Robust regression was used to fit the model, and stepwise regression and variance inflation coefficient (VIF < 5) were used to exclude overfitting of the model and reduce variable collinearity. The obtained “optimal model” included predictive variables such as Pwet, Twet, NPP, N deposition, Soil P, Clay, and ALSA, with the explanation of R_adj_^2^=0.59. Elasticity of our model statistics (homoscedasticity, multivariate normality, independence of residuals, and low Multicollinearity) meets regression analysis diagnosis (Figure S9). And a 1:1 fit between the fitted value and the observed value was also exhibited (Figure S10).


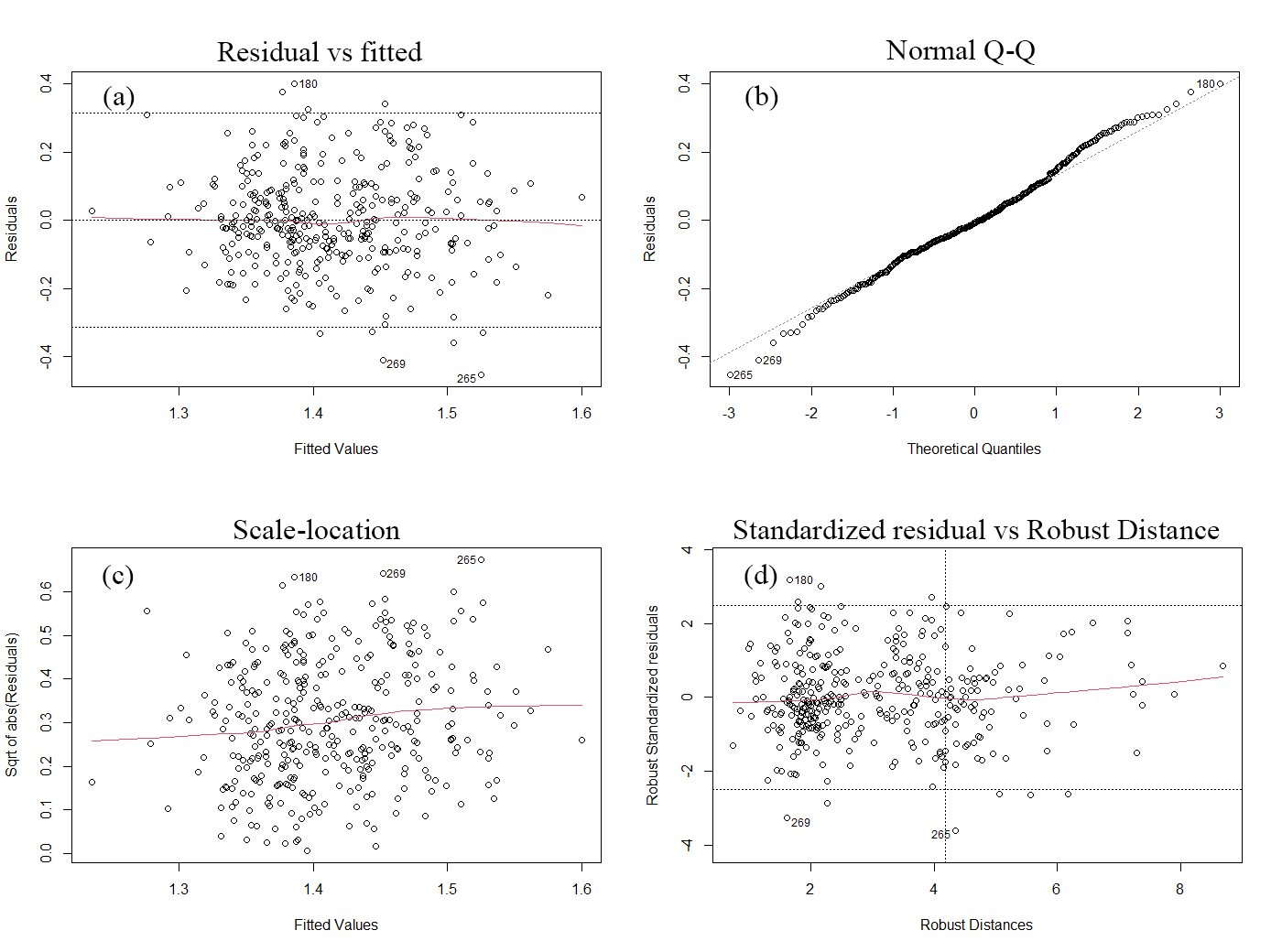


**Figure S9** Diagnostic plots for the best lmrob model. The Residuals vs Fitted plot shows an approximate horizontal line, without distinct patterns, indicating that a linear relationship is appropriate for our analysis. The normal Q-Q plot indicates that our model residuals are normally distributed. The scale location plot indicates relative homogeneity of variance of the residuals (homoscedasticity). The standardized residuals vs Robust Distance Plot indicates that there is no obvious outlier may affect model results.


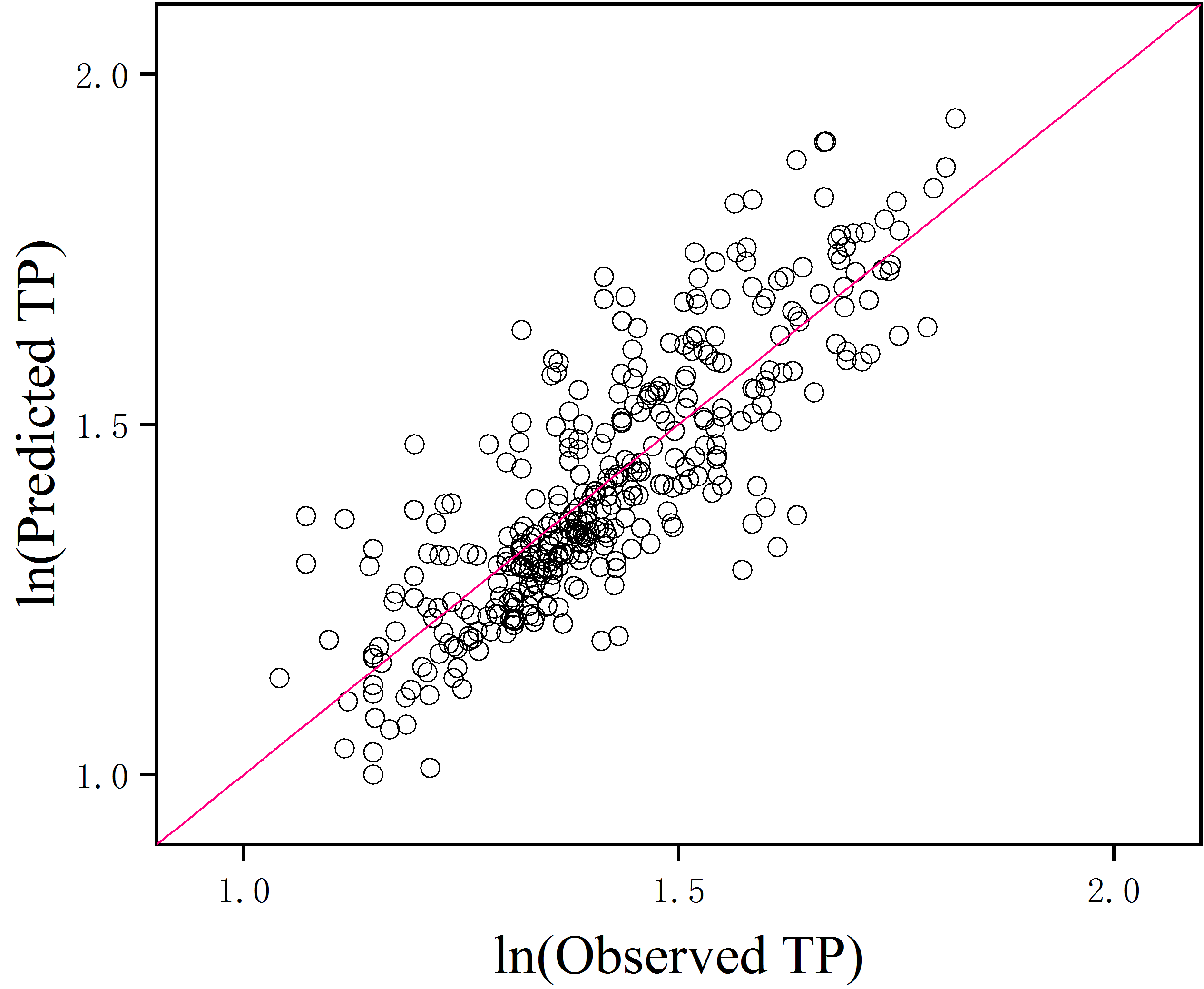


**Figure S10** Comparison of observed vs. predicted total P deposition. Red line represents the 1:1 line. Each blank circle represents an individual observation.

**Table S4** Summary of the sources of plant traits extracted from TRY plant trait (TRY) database

| **Database ID from TRY** | **Number of observation** | **Reference** |
| --- | --- | --- |
| 221 | 7 | (Wright *et al.*, 2004) |
| 443 | 415 | (Wang *et al.*, 2017b) |
| 412 | 2131 | (Vergutz *et al.*, 2012) |
| 331 | 118 | (van der Plas & Olff, 2014) |
| 333 | 52 | (van de Weg *et al.*, 2009) |
| 288 | 83 | (Swenson *et al.*, 2011) |
| 94 | 38 | (Reich *et al.*, 2009) |
| 236 | 684 | (Prentice *et al.*, 2011) |
| 91 | 79 | (Ogaya & Peñuelas, 2003) |
| 290 | 40 | (Minden & Kleyer, 2014) |
| 342 | 6 | (Maire *et al.*, 2015) |
| 193 | 165 | (Laughlin *et al.*, 2011) |
| 352 | 231 | (Kearsley *et al.*, 2017) |
| 50 | 10 | (Kazakou *et al.*, 2006) |
| 310 | 120 | (Gos *et al.*, 2016) |
| 45 | 197 | (Garnier *et al.*, 2007) |
| 34 | 359 | (Fyllas *et al.*, 2009) |
| 105 | 30 | (Freschet *et al.*, 2010) |
| 377 | 216 | (Chacon-Madrigal *et al.*, 2018) |
| 114 | 1 | (Dunbar-Co *et al.*, 2009) |
| 231 | 241 | (Domingues *et al.*, 2010) |
| 230 | 22 | (Craven *et al.*, 2007) |
| 130 | 2 | (Craine *et al.*, 2009) |
| 295 | 13 | (Blonder *et al.*, 2016) |
| 422 | 5 | (Baruch & Goldstein, 1999) |
| 269 | 459 | (Baraloto *et al.*, 2010) |
| 292 | 2 | (Aubin et al., 2012) |
| 97 | 2 | (Atkin *et al.*, 2015) |
| 285 | 9 | (Adler *et al.*, 2004) |

Table S5 Summary of atmospheric P deposition of 125 sites added in this study.

| **Region** | **site** | **Sampling time** | **Wet deposition** | **Dry deposition** | **Bulk** | **PO_4_-P** | **Reference** |
| --- | --- | --- | --- | --- | --- | --- | --- |
| Africa | Ras Younga | 2014—2015 |  |  | 42 |  | (Khammeri *et al.*, 2018) |
|  | Bujumbura | 1994—1995 | 130 |  |  |  | (Langenberg *et al.*, 2003) |
|  | Kigoma | 1994—1995 | 48 |  |  |  | (Langenberg *et al.*, 2003) |
|  | Mpulungu | 1994—1997 | 25 |  |  |  | (Langenberg *et al.*, 2003) |
|  | Quéntar reservoir | 2004—2005 |  |  | 61 |  | (Morales-Baquero & Perez-Martinez, 2016) |
| Asia | Daya bay | 2012—2012 |  | 14.64 | 21.64 |  | (Chen *et al.*, 2014) |
|  | Jilong | 2003—2004 |  |  | 16 |  | (Chen *et al.*, 2008) |
|  | Jilong | 2003—2004 |  | 16 |  |  | (Chen *et al.*, 2008) |
|  | Lake Baikal | 1994—1995 | 20 |  |  |  | (Callender & Granina, 1997) |
|  | R1 | 2009—2018 | 21 |  |  |  | (Chiwa, 2020) |
|  | R2 | 2009—2018 | 12 |  |  |  | (Chiwa, 2020) |
|  | S | 2009—2018 | 28 |  |  |  | (Chiwa, 2020) |
|  | Small Catchment Around Miyun Reservoir | 2019—2020 | 70 | 125 | 206 |  | (Chen *et al.*, 2022) |
|  | Yangquangou station | 2015—2015 |  |  | 21 |  | (Chen *et al.*, 2017) |
|  | Xiangxi River | 2014—2015 | 60 |  |  |  | (Gao *et al.*, 2017) |
|  | Beili Lake | 2011—2012 | 23 | 64 | 89 |  | (Gu *et al.*, 2013) |
|  | Kutsuki | 1993—1999 |  |  | 66 |  | (Hartmann *et al.*, 2008) |
|  | Kutsuki | 2000—2005 |  |  | 126 |  | (Hartmann *et al.*, 2008) |
|  | Marina | 2007—2008 |  |  | 191 |  | (He *et al.*, 2011) |
|  | Shanghai | 2005—2005 | 1 |  |  |  | (Huang *et al.*, 2008) |
|  | Taihu Basin | 2013—2014 |  |  | 26 |  | (Liu, 2018) |
|  | Dahekou reservoir | 2014—2014 | 105.24 | 170.28 | 275.52 |  | (Lu & Tian, 2017) |
|  | Fengqiu | 1994—1995 | 3 |  |  |  | (Lu *et al.*, 1996) |
|  | Dinghushan | 1997—1999 | 101.5 |  |  |  | (Mo *et al.*, 2002) |
|  | Beijing | 2013—2014 | 6 |  |  |  | (Ouyang *et al.*, 2019) |
|  | Kubu Raya District | 2013—2014 |  |  | 79 |  | (Ponette-Gonzalez *et al.*, 2016) |
|  | Kumaun Himalaya (2050 masl) | 1981—1982 |  |  | 100 |  | (Pandey *et al.*, 1983) |
|  | East Lake in Wuhan | 2017—2018 |  |  | 137 |  | (Peng *et al.*, 2019) |
|  | Rizegou of Jiuzhaigou | 2011—2012 |  |  | 22 |  | (Qiao *et al.*, 2014) |
|  | Goukou of Jiuzhaigou | 2010—2011 |  |  | 21 |  | (Qiao *et al.*, 2014) |
|  | Lake Dianchi | 2014—2014 |  |  | 14 |  | (Ren *et al.*, 2019) |
|  | chengdu | 2008—2018 |  |  | 42 |  | (Song *et al.*, 2022) |
|  | Gongga Mountains | 2007—2014 |  |  | 5 |  | (Song *et al.*, 2022) |
|  | Shifang | 2015—2018 |  |  | 88 |  | (Song *et al.*, 2022) |
|  | Yanting town | 2008—2018 |  |  | 26 |  | (Song *et al.*, 2022) |
|  | Changshu Ecological Station | 2003—2004 | 110 |  |  |  | (Wang *et al.*, 2009) |
|  | Daya Bay | 2015—2017 | 12 |  |  |  | (Wu *et al.*, 2021) |
|  | Daya Bay | 2015—2017 |  |  | 3 |  | (Wu *et al.*, 2018) |
|  | Hangzhou | 2013—2014 | 20.3 | 45.2 | 65.2 |  | (Wang *et al.*, 2015a) |
|  | Huzhou | 2013—2014 | 27.2 | 40.1 | 67.2 |  | (Wang *et al.*, 2015a) |
|  | Jiaxing | 2013—2014 | 22.7 | 46.9 | 69.7 |  | (Wang *et al.*, 2015a) |
|  | Taihu | 2007—2010 | 172 |  |  |  | (Wang *et al.*, 2017a) |
|  | Northwest suburb of Hefei | 2014—2016 |  |  | 67 |  | (Wei *et al.*, 2018) |
|  | Jiaozhou Bay | 2015—2016 |  |  | 8 |  | (Xing *et al.*, 2018) |
|  | Miyun Reservoir | 1991—1999 |  |  | 182 |  | (Xu *et al.*, 2003) |
|  |  |  |  |  |  |  |  |
|  | Qianyanzhou Station | 2013—2014 | 38 |  |  |  | (Xu *et al.*, 2016) |
|  | Shennongjia (Hongpin Town) | 2015—2015 |  |  | 28 |  | (Yang *et al.*, 2018) |
|  | Lake Yangzonghai | 2012—2014 |  |  | 24 |  | (Yu *et al.*, 2017) |
|  | Taihu | 2002—2003 | 85 | 311 | 395 |  | (Zhai *et al.*, 2009) |
|  | Shanghai | 2007—2008 | 38 |  |  |  | (Zhang *et al.*, 2011) |
|  | Shanghai | 2007—2008 | 32 |  |  |  | (Zhang *et al.*, 2011) |
|  | Qianliyan Island | 1998—1998 | 23 |  |  |  | (Zhang *et al.*, 2000) |
|  | Lake Qinghai | 2017—2018 |  |  | 186 |  | (Zhang *et al.*, 2019) |
|  | Lake Qinghai | 2017—2018 | 186 |  |  |  | (Zhang *et al.*, 2019) |
|  | Laoshan | 1992—1993 | 25 |  |  |  | (Zhang *et al.*, 1999) |
|  | Qianliyan Island | 1992—1993 | 36 |  |  |  | (Zhang *et al.*, 1999) |
|  | Qingdao | 1992—1993 | 83 |  |  |  | (Zhang *et al.*, 1999) |
|  | Qingdao Coast | 1998—1998 | 5 |  |  |  | (Zhang *et al.*, 1999) |
|  | Longnan | 1979—1980 | 83 |  |  |  | (Zhang & Wei, 1992) |
|  | Qiandao Lake | 2020—2021 | 18 | 16 | 37 |  | (Zhu *et al.*, 2022) |
|  | Zhoushan | 2008—2009 | 2 |  |  |  | (Zhu & Liu, 2011) |
|  | Beili Lake | 2011—2012 | 81 | 18 | 100 |  | (Zhu *et al.*, 2015) |
|  | Liuxihe National | 2006—2008 | 26 |  |  |  | (Zhou, Tian and Yang, 2009) |
|  | Zhoucun reservoir | 2016—2017 | 20.127 |  |  |  | (Zhou *et al.*, 2020) |
|  | Kaihua County | 2021—2021 |  |  | 53 |  | (Zhou *et al.*, 2022) |
| Europe | Zingst | 2013—2017 |  |  | 26 |  | (Berthold *et al.*, 2019) |
|  | St Nicholas Valley | 1990—1990 |  |  | 60.6298 |  | (Camerero and Catalan, 1996) |
|  | South lake district (Merlewood) | 1962—1965 |  |  | 100 |  | (Carlisle *et al.*, 1967) |
|  | Frioul Island | 2015—2016 |  |  | 3 |  | (Djaoudi *et al.*, 2018) |
|  | Alps | 1989—1991 |  | 7.3 |  |  | (Deangelis & Gaudichet, 1991) |
|  | Aliaga | 2014—2015 |  |  | 25 |  | (Filizok and Gorgun, 2019) |
|  | Bornova | 2014—2015 |  |  | 21 |  | (Filizok & Gorgun, 2019) |
|  | Dikili | 2014—2015 |  |  | 15 |  | (Filizok & Gorgun, 2019) |
|  | North | 1998—2000 |  |  | 24.2 |  | (Knulst, 2001) |
|  | Skalnaté Pleso | 1998—2009 |  |  | 14 |  | (Kopacek *et al.*, 2011) |
|  | Plešné Lake | 1998—2009 |  |  | 22 |  | (Kopacek *et al.*, 2011) |
|  | Certovo Lake | 1998—2009 |  |  | 28 |  | (Kopacek *et al.*, 2011) |
|  | Slapy Reservoir | 1998—2009 |  |  | 16 |  | (Kopacek *et al.*, 2011) |
|  | Starolesniaske Pleso | 1998—2009 |  |  | 26 |  | (Kopacek *et al.*, 2011) |
|  | Tatra Mountains | 1998—2009 |  |  | 21.452 |  | (Kopacek *et al.*, 2011) |
|  | Varna | 2012—2014 |  |  |  | 51 | (Kocak *et al.*, 2016) |
|  | 07FRII Northeast | 1998—1999 |  |  | 16.337 |  | (Mosello *et al.*, 2002) |
|  | 08FR12 Northeast | 1998—1999 |  |  | 4.236 |  | (Mosello *et al.*, 2002) |
|  | 10LOMI Northeast | 1998—1999 |  |  | 18.75 |  | (Mosello *et al.*, 2002) |
|  | 17TREI Northeast | 1998—1999 |  |  | 113.568 |  | (Mosello *et al.*, 2002) |
|  | 20VENI Northeast | 1998—1999 |  |  | 6.835 |  | (Mosello *et al.*, 2002) |
|  | Cap Ferrat | 1998—1998 |  | 5 |  |  | (Migon *et al.*, 2001) |
|  | Sierra Nevada | 2001—2002 |  |  | 15.8999 |  | (Morales-Baquero *et al.*, 2006) |
|  | Warren Farm | 2002—2003 |  |  | 19 |  | (Neal *et al.*, 2004) |
|  | Alps | 1983—1986 |  |  | 7.6 |  | (Psenner, 1989) |
|  | Black Forest | 2015—2016 |  |  | 17 |  | (Sohrt *et al.*, 2019) |
|  | Birkenes South (BI) | 1998—1998 |  |  | 22.6176 |  | (Saltbones *et al.*, 1998) |
|  | Sogne South (SG) | 1998—1998 |  |  | 20.8692 |  | (Saltbones *et al.*, 1998) |
|  | Hurdal Central (HU) | 1998—1998 |  |  | 14.8025 |  | (Saltbones *et al.*, 1998) |
|  | Langtjern Central (LA) | 1998—1998 |  |  | 18.0544 |  | (Saltbones *et al.*, 1998) |
|  | Fagernes Central (FA) | 1998—1998 |  |  | 13.1068 |  | (Saltbones *et al.*, 1998) |
|  | Valle South (VA) | 1998—1998 |  |  | 15.7852 |  | (Saltbones *et al.*, 1998) |
|  | University of Tehran | 2013—2014 |  |  | 80 |  | (Salehi *et al.*, 2016) |
|  | St Nicholas Valley | 1997—1998 |  |  | 8.73 |  | (Ventura *et al.*, 2000) |
|  | Cap Béar | 2012—2013 |  |  | 67 |  | (Violaki *et al.*, 2018) |
|  | Finokalia | 2005—2011 |  |  | 46 |  | (Violaki *et al.*, 2018) |
| North America | South Flora | 1992—1996 |  | 31 |  |  | (Ahn & James, 1999) |
|  | South Florida | 1992—1996 | 16 |  |  |  | (Ahn & James, 1999) |
|  | South Florida | 1974—1999 | 11 |  |  |  | (Ahn & James, 2001) |
|  | Cordillera Real (Montane) | 1998—2003 |  |  | 93 |  | (Boy & Wilcke, 2008) |
|  | Montane (Mean) | 1998—2006 |  |  | 137.7 |  | (Boy & Wilcke, 2008) |
|  | N. Wind River Range, Wyoming | 2009—2010 |  |  | 15 |  | (Brahney, 2012) |
|  | S. Wind River Range, Wyoming | 2009—2010 |  |  | 38 |  | (Brahney, 2012) |
|  | San Juan Mountains, Colorado | 2009—2010 |  |  | 18 |  | (Brahney, 2012) |
|  | Boston | 2015—2015 |  |  | 77 |  | (Decina *et al.*, 2018) |
|  | Podocarpus National Park | 2002—2006 | 260 |  |  |  | (Fabian *et al.*, 2009) |
|  | Florida | 1992—1993 | 1 |  |  |  | (Grimshaw & Dolske, 2002) |
|  | Torres del Paine (Patagonia) | 1984—1993 | 13.4 |  |  |  | (Galloway *et al.*, 1996) |
|  | Turrialba | 1979—1981 |  |  | 20 |  | (Hendry *et al.*, 1984) |
|  | Lake Tahoe | 1989—1991 |  |  | 33 |  | (Jassby *et al.*, 1994) |
|  | Hubbard Brook, New Hampshire | 1972—2010 |  |  | 17 |  | Likens (1995-2010), Hubbard Brook Dataset |
|  | Cienfuegos | 2014—2016 |  |  | 38 |  | (Morera-Gomez *et al.*, 2019) |
|  | Colorado Rockies, Green Lake valley | 2002—2010 |  |  | 15 |  | (Mladenov *et al.*, 2012) |
|  | Banff National Park | 2008—2008 | 57.3 |  |  |  | (Porter, 2012) |
|  | Florida | 1992—1996 | 8 |  |  |  | (Pollman *et al.*, 2002) |
|  | Lake Tahoe, California | 1994—2004 |  |  | 19 |  | (Sahoo *et al.*, 2010) |
|  | Syracuse | 2010—2011 |  |  | 48 |  | (Todorov *et al.*, 2018) |
|  | Vander (Montane) | 1986—1987 |  |  | 60 |  | (Veneklaas, 1990) |
|  | Sequoia National Park | 2008—2008 |  | 15 |  |  | (Vicars *et al.*, 2010) |

**Reference**

Adler PB, Milchunas DG, Lauenroth WK, Sala OE, Burke IC (2004) Functional traits of graminoids in semi-arid steppes: a test of grazing histories. Journal of Applied Ecology*,* **41**, 653-663.

Ahn H, James RT (2001) Variability, uncertainty, and sensitivity of phosphorus deposition load estimates in South Florida. Water Air and Soil Pollution*,* **126**, 37-51.

Ahn HS, James RT (1999) Outlier detection in phosphorus dry deposition rates measured in South Florida. Atmospheric Environment*,* **33**, 5123-5131.

Atkin OK, Bloomfield KJ, Reich PB *et al.* (2015) Global variability in leaf respiration in relation to climate, plant functional types and leaf traits. New Phytologist*,* **206**, 614-636.

Baraloto C, Paine CET, Poorter L *et al.* (2010) Decoupled leaf and stem economics in rain forest trees. Ecology Letters*,* **13**, 1338-1347.

Baruch Z, Goldstein G (1999) Leaf construction cost, nutrient concentration, and net CO2 assimilation of native and invasive species in Hawaii. Oecologia*,* **121**, 183-192.

Batjes (2015) World soil property estimates for broad-scale modelling (WISE30sec). Report 2015/01, ISRIC - World Soil Information, Wageningen (with data set, available at <www.isric.org>).

Berthold M, Wulff R, Reiff V, Karsten U, Nausch G, Schumann R (2019) Magnitude and influence of atmospheric phosphorus deposition on the southern Baltic Sea coast over 23years: implications for coastal waters. Environmental Sciences Europe*,* **31**.

Blonder B, Baldwin BG, Enquist BJ, Robichaux RH (2016) Variation and macroevolution in leaf functional traits in the Hawaiian silversword alliance (Asteraceae). Journal of Ecology*,* **104**, 219-228.

Boy J, Wilcke W (2008) Tropical Andean forest derives calcium and magnesium from Saharan dust. Global Biogeochemical Cycles*,* **22**.

Callender E, Granina L (1997) Biogeochemical phosphorus mass balance for Lake Baikal, southeastern Siberia, Russia. Marine Geology*,* **139**, 5-19.

Carlisle A, Brown AHF, White EJ (1967) The nutrient content of tree stem flow and ground flora litter and leachates in a sessile oak (Quercus Petraea) woodland. Journal of Ecology*,* **55**, 615-+.

Chacon-Madrigal E, Wanek W, Hietz P, Dullinger S (2018) Traits indicating a conservative resource strategy are weakly related to narrow range size in a group of neotropical trees. Perspectives in Plant Ecology Evolution and Systematics*,* **32**, 30-37.

Chen H-Y, Hung C-C, Fang T-H, Gong G-C (2008) Dry deposition and particle-size distribution of phosphorus in the marine atmosphere over the northeastern coast of Taiwan. Continental Shelf Research*,* **28**, 756-766.

Chen H, Wang X, Huang J, Huang J, Nan Z, Ren J (2022) Dry and wet atmospheric deposition of nitrogen and phosphorus in small catchment around miyun reservoir. Research of Environmental Sciences*,* **35**, 1419-1431 (in chinese).

Chen J, Lu P, Chen Z, Yan H, Li L (2014) Atmospheric deposition of nitrogen and phosphorus at Daya Bay in Huizhou during spring and summer. Journal of Tropical Oceanography*,* **33**, 109-114 (in chinese).

Chen S, Wang Y, Gao Y, Jia J, Chen W, Chen L (2017) Characteristics of phosphorus wet deposition in the loess plateau and its effect on the phosphorus export in the dam watershed: A case study of Yangjuangou watershed. Acta Scientiae Circumstantiae*,* **37**, 721-728.

Chiwa M (2020) Ten-year determination of atmospheric phosphorus deposition at three forested sites in Japan. Atmospheric Environment*,* **223**.

Craine JM, Elmore AJ, Aidar MPM *et al.* (2009) Global patterns of foliar nitrogen isotopes and their relationships with climate, mycorrhizal fungi, foliar nutrient concentrations, and nitrogen availability. New Phytologist*,* **183**, 980-992.

Craven D, Braden D, Ashton MS, Berlyn GP, Wishnie M, Dent D (2007) Between and within-site comparisons of structural and physiological characteristics and foliar nutrient content of 14 tree species at a wet, fertile site and a dry, infertile site in Panama. Forest Ecology and Management*,* **238**, 335-346.

Deangelis M, Gaudichet A (1991) Saharan dust deposition over Mont-Blanc (Ferench alps) duration the last 30 years

Tellus Series B-Chemical and Physical Meteorology*,* **43**, 61-75.

Decina SM, Templer PH, Hutyra LR (2018) Atmospheric Inputs of Nitrogen, Carbon, and Phosphorus across an Urban Area: Unaccounted Fluxes and Canopy Influences. Earths Future*,* **6**, 134-148.

Djaoudi K, Van Wambeke F, Barani A, Helias-Nunige S, Sempere R, Pulido-Villena E (2018) Atmospheric fluxes of soluble organic C, N, and P to the Mediterranean Sea: Potential biogeochemical implications in the surface layer. Progress in Oceanography*,* **163**, 59-69.

Domingues TF, Meir P, Feldpausch TR *et al.* (2010) Co-limitation of photosynthetic capacity by nitrogen and phosphorus in West Africa woodlands. Plant Cell and Environment*,* **33**, 959-980.

Dunbar-Co S, Sporck MJ, Sack L (2009) Leaf trait diversification and design in seven rare taxa of the Hawiian plantago radiation. International Journal of Plant Sciences*,* **170**, 61-75.

Fabian P, Rollenbeck R, Spichtinger N, Dominguez G, Brothers L, Thiemens M (2009) Desert dust,Ocean spray,Volcanoes,Biomass burning: Pathways of nutrients into Andean rainforests. Advances in Geosciences*,* **11**, 2081.

Fick SE, Hijmans RJ (2017) WorldClim 2: new 1-km spatial resolution climate surfaces for global land areas. International Journal of Climatology*,* **37**, 4302-4315.

Filizok I, Gorgun AU (2019) Atmospheric depositional characteristics of Po-210, Pb-210 and some trace elements in Izmir, Turkey. Chemosphere*,* **220**, 468-475.

Freschet GT, Cornelissen JHC, Van Logtestijn RSP, Aerts R (2010) Evidence of the 'plant economics spectrum' in a subarctic flora. Journal of Ecology*,* **98**, 362-373.

Fyllas NM, Patino S, Baker TR *et al.* (2009) Basin-wide variations in foliar properties of Amazonian forest: phylogeny, soils and climate. Biogeosciences*,* **6**, 2677-2708.

Galloway JN, Keene WC, Likens GE (1996) Processes controlling the composition of precipitation at a remote southern hemispheric location: Torres del Paine National Park, Chile. Journal of Geophysical Research-Atmospheres*,* **101**, 6883-6897.

Gao Y, Hao Z, Yang T, He N, Wen X, Yu G (2017) Effects of atmospheric reactive phosphorus deposition on phosphorus transport in a subtropical watershed: A Chinese case study. Environmental Pollution*,* **226**, 69-78.

Garnier E, Lavorel S, Ansquer P *et al.* (2007) Assessing the effects of land-use change on plant traits, communities and ecosystem functioning in grasslands: A standardized methodology and lessons from an application to 11 European sites. Annals of Botany*,* **99**, 967-985.

Gos P, Loucougaray G, Colace M-P *et al.* (2016) Relative contribution of soil, management and traits to co-variations of multiple ecosystem properties in grasslands. Oecologia*,* **180**, 1001-1013.

Grimshaw HJ, Dolske DA (2002) Rainfall concentrations and wet atmospheric deposition of phosphorus and other constituents in Florida, USA. Water Air and Soil Pollution*,* **137**, 117-140.

Gu D, Deng K, Li T, Jiao F (2013) Study on the atmospheric deposition of nitrogen and phosphorus in Beili Lake hangzhou. Safe and Environmental Engineering*,* **20**, 36-40 (in chinese).

Hartmann J, Kunimatsu T, Levy JK (2008) The impact of Eurasian dust storms and anthropogenic emissions on atmospheric nutrient deposition rates in forested Japanese catchments and adjacent regional seas. Global and Planetary Change*,* **61**, 117-134.

He J, Balasubramanian R, Burger DF, Hicks K, Kuylenstierna JCI, Palani S (2011) Dry and wet atmospheric deposition of nitrogen and phosphorus in Singapore. Atmospheric Environment*,* **45**, 2760-2768.

Hendry CD, Berish CW, Edgerton ES (1984) PRECIPITATION CHEMISTRY AT TURRIALBA, COSTA-RICA. Water Resources Research*,* **20**, 1677-1684.

Huang K, Zhuang G, Xu C, Wang Y, Tang A (2008) The chemistry of the severe acidic precipitation in Shanghai, China. Atmospheric Research*,* **89**, 149-160.

Jassby AD, Reuter JE, Axler RP, Goldman CR, Hackley SH (1994) ATMOSPHERIC DEPOSITION OF NITROGEN AND PHOSPHORUS IN THE ANNUAL NUTRIENT LOAD OF LAKE TAHOE (CALIFORNIA NEVADA). Water Resources Research*,* **30**, 2207-2216.

Kazakou E, Vile D, Shipley B, Gallet C, Garnier E (2006) Co-variations in litter decomposition, leaf traits and plant growth in species from a Mediterranean old-field succession. Functional Ecology*,* **20**, 21-30.

Kearsley E, Verbeeck H, Hufkens K *et al.* (2017) Functional community structure of African monodominant Gilbertiodendron dewevrei forest influenced by local environmental filtering. Ecology and Evolution*,* **7**, 295-304.

Khammeri Y, Hamza IS, Zouari AB *et al.* (2018) Atmospheric bulk deposition of dissolved nitrogen, phosphorus and silicate in the Gulf of Gabes (South Ionian Basin); implications for marine heterotrophic prokaryotes and ultraphytoplankton. Continental Shelf Research*,* **159**, 1-11.

Kocak M, Mihalopoulos N, Tutsak E, Violaki K, Theodosi C, Zarmpas P, Kalegeri P (2016) Atmospheric Deposition of Macronutrients (Dissolved Inorganic Nitrogen and Phosphorous) onto the Black Sea and Implications on Marine Productivity*. Journal of the Atmospheric Sciences*,* **73**, 1727-1739.

Kopacek J, Hejzlar J, Vrba J, Stuchlik E (2011) Phosphorus loading of mountain lakes: Terrestrial export and atmospheric deposition. Limnology and Oceanography*,* **56**, 1343-1354.

Langenberg VT, Nyamushahu S, Roijackers R, Koelmans AA (2003) External nutrient sources for Lake Tanganyika. Journal of Great Lakes Research*,* **29**, 169-180.

Laughlin DC, Fule PZ, Huffman DW, Crouse J, Laliberte E (2011) Climatic constraints on trait-based forest assembly. Journal of Ecology*,* **99**, 1489-1499.

Liu L (2018) Study on atmospheric sedimentation flux in Taihu Lake of Jiangsu province. China Resources Comprehensive Utilization*,* **36**, 175-179 (in chinese).

Lu C, Tian H (2017) Global nitrogen and phosphorus fertilizer use for agriculture production in the past half century: shifted hot spots and nutrient imbalance. Earth System Science Data*,* **9**, 181-192.

Lu R, Liu H, Wen D, Qin S, Zheng J, Wang Z (1996) Nitrogen cycling and balance in the typical agricultural area in China. Journal of Soil Science.

Mahowald N, Jickells TD, Baker AR *et al.* (2008) Global distribution of atmospheric phosphorus sources, concentrations and deposition rates, and anthropogenic impacts. Global Biogeochemical Cycles*,* **22**.

Maire V, Wright IJ, Prentice IC *et al.* (2015) Global effects of soil and climate on leaf photosynthetic traits and rates. Global Ecology and Biogeography*,* **24**, 706-717.

Migon C, Sandroni V, Bethoux JP (2001) Atmospheric input of anthropogenic phosphorus to the northwest Mediterranean under oligotrophic conditions. Marine Environmental Research*,* **52**, 413-426.

Minden V, Kleyer M (2014) Internal and external regulation of plant organ stoichiometry. Plant Biology*,* **16**, 897-907.

Mladenov N, Williams MW, Schmidt SK, Cawley K (2012) Atmospheric deposition as a source of carbon and nutrients to an alpine catchment of the Colorado Rocky Mountains. Biogeosciences*,* **9**, 3337-3355.

Mo J, Fang Y, Zhang D, Kong G, Feng Z (2002) Effects of rainfall reallocation on nutrient dynamic of a pine forest in Dinghushan. Guihaia*,* **22**, 529-533,536 (in chinese).

Morales-Baquero R, Perez-Martinez C (2016) Saharan versus local influence on atmospheric aerosol deposition in the southern Iberian Peninsula: Significance for N and P inputs. Global Biogeochemical Cycles*,* **30**, 501-513.

Morales-Baquero R, Pulido-Villena E, Reche I (2006) Atmospheric inputs of phosphorus and nitrogen to the southwest Mediterranean region: Biogeochemical responses of high mountain lakes. Limnology and Oceanography*,* **51**, 830-837.

Morera-Gomez Y, Miguel Santamaria J, Elustondo D, Lasheras E, Manuel Alonso-Hernandez C (2019) Determination and source apportionment of major and trace elements in atmospheric bulk deposition in a Caribbean rural area. Atmospheric Environment*,* **202**, 93-104.

Mosello R, Maria C, Brizzio D, Kotzias DD, Marchetto A, Rembges D, Tartari G (2002) The chemistry of atmospheric deposition in Italy in the framework of the National Programme for Forest Ecosystems Control (CONECOFOR). J. Limnol*,* **61**, 77-92.

Neal C, Skeffington R, Neal M, Wyatt R, Wickham H, Hill L, Hewitt N (2004) Rainfall and runoff water quality of the Pang and Lambourn, tributaries of the River Thames, south-eastern England. Hydrology and Earth System Sciences*,* **8**, 601-613.

Ogaya R, Peñuelas J (2003) Comparative field study of Quercus ilex and Phillyrea latifolia: photosynthetic response to experimental drought conditions. Environmental and Experimental Botany*,* **50**, 137-148.

Ouyang W, Xu Y, Cao J, Gao X, Gao B, Hao Z, Lin C (2019) Rainwater characteristics and interaction with atmospheric particle matter transportation analyzed by remote sensing around Beijing. Science of the Total Environment*,* **651**, 532-540.

Pan Y, Liu B, Cao J, Liu J, Tian S, Du E (2021) Enhanced atmospheric phosphorus deposition in Asia and Europe in the past two decades. Atmospheric and Oceanic Science Letters*,* **14**.

Pandey AN, Pathak PC, Singh JS (1983) Water, sediment and nutrient movement in forested and non-forested catchements in Kumaun Himalaya. Forest Ecology and Management*,* **7**, 19-29.

Peng Q, Li Z, Deng X, Su M (2019) Nitrogen and phosphorus deposition in urban lakes and its impact factors: A case study of East Lake in Wuhan. Acta Scientiae Circumstantiae*,* **39**, 2635-2643 (in chinese).

Pollman CD, Landing WM, Perry JJ, Fitzpatrick T (2002) Wet deposition of phosphorus in Florida. Atmospheric Environment*,* **36**, 2309-2318.

Ponette-Gonzalez AG, Curran LM, Pittman AM *et al.* (2016) Biomass burning drives atmospheric nutrient redistribution within forested peatlands in Borneo. Environmental Research Letters*,* **11**.

Prentice IC, Meng T, Wang H, Harrison SP, Ni J, Wang G (2011) Evidence of a universal scaling relationship for leaf CO2 drawdown along an aridity gradient. New Phytologist*,* **190**, 169-180.

Psenner R (1989) Chemistry of high mountain lakes in siliceous catchments of the centural eastern alps. Aquatic Sciences*,* **51**, 108-128.

Qiao X, Jiang L, Tang Y, Xiong F, Du J, Xiao W (2014) The Fluxes and Possible Aquatic Impacts of Atmospheric Nitrogen,Sulfur and Phosphorous Deposition in Jiuzhaigou. Mountain Research*,* **32**, 633-640 (in chinese).

Reich PB, Oleksyn J, Wright IJ (2009) Leaf phosphorus influences the photosynthesis-nitrogen relation: a cross-biome analysis of 314 species. Oecologia*,* **160**, 207-212.

Ren J-G, Jia H-B, Jiao L-X *et al.* (2019) Characteristics of Nitrogen and Phosphorus Formation in Atmospheric Deposition in Dianchi Lake and Their Contributions to Lake Loading. Huan jing ke xue= Huanjing kexue*,* **40**, 582-589 (in chinese).

Sahoo GB, Schladow SG, Reuter JE (2010) Effect of sediment and nutrient loading on Lake Tahoe optical conditions and restoration opportunities using a newly developed lake clarity model. Water Resources Research*,* **46**.

Salehi M, Amiri GZ, Attarod P, Salehi A, Brunner I, Schleppi P, Thimonier A (2016) Seasonal variations of throughfall chemistry in pure and mixed stands of Oriental beech (Fagus orientalis Lipsky) in Hyrcanian forests (Iran). Annals of Forest Science*,* **73**, 371-380.

Saltbones J, Foss A, Bartnicki J (1998) Norwegian Meteorological Institute's real-time dispersion model SNAP (severe nuclear accident program): Runs for ETEX and ATMES II experiments with different meteorological input. Atmospheric Environment*,* **32**, 4277-4283.

Sohrt J, Uhlig D, Kaiser K *et al.* (2019) Phosphorus Fluxes in a Temperate Forested Watershed: Canopy Leaching, Runoff Sources, and In-Stream Transformation. Frontiers in Forests and Global Change*,* **2**.

Song L, Kuang F, Zhou M, Zhu B, Skiba U (2022) Bulk phosphorous deposition at four typical land use sites in Southwest China. Chemosphere*,* **288**.

Swenson NG, Anglada-Cordero P, Barone JA (2011) Deterministic tropical tree community turnover: evidence from patterns of functional beta diversity along an elevational gradient. Proceedings of the Royal Society B-Biological Sciences*,* **278**, 877-884.

Tipping E, Benham S, Boyle JF *et al.* (2014) Atmospheric deposition of phosphorus to land and freshwater. Environ Sci Process Impacts*,* **16**, 1608-1617.

Todorov D, Driscoll CT, Todorova S, Montesdeoca M (2018) Water quality function of an extensive vegetated roof. Science of the Total Environment*,* **625**, 928-939.

Van De Weg MJ, Meir P, Grace J, Atkin OK (2009) Altitudinal variation in leaf mass per unit area, leaf tissue density and foliar nitrogen and phosphorus content along an Amazon-Andes gradient in Peru. Plant Ecology & Diversity*,* **2**, 243-U247.

Van Der Plas F, Olff H (2014) Mesoherbivores affect grasshopper communities in a megaherbivore-dominated South African savannah. Oecologia*,* **175**, 639-649.

Veneklaas EJ (1990) NUTRIENT FLUXES IN BULK PRECIPITATION AND THROUGHFALL IN 2 MONTANE TROPICAL RAIN-FORESTS, COLOMBIA. Journal of Ecology*,* **78**, 974-992.

Ventura M, Camarero L, Buchaca T, Bartumeus F, Livingstone D, Catalan J (2000) The main features of seasonal variability in the external forcing and dynamics of a deep mountain lake (Redó, Pyrenees). Journal of Limnology*,* **59**, 97-108.

Vergutz L, Manzoni S, Porporato A, Novais RF, Jackson RB (2012) Global resorption efficiencies and concentrations of carbon and nutrients in leaves of terrestrial plants. Ecological Monographs*,* **82**, 205-220.

Vicars WC, Sickman JO, Ziemann PJ (2010) Atmospheric phosphorus deposition at a montane site: Size distribution, effects of wildfire, and ecological implications. Atmospheric Environment*,* **44**, 2813-2821.

Violaki K, Bourrin F, Aubert D, Kouvarakis G, Delsaut N, Mihalopoulos N (2018) Organic phosphorus in atmospheric deposition over the Mediterranean Sea: An important missing piece of the phosphorus cycle. Progress in Oceanography*,* **163**, 50-58.

Wang J, Zhao Q, Pang Y, Hu K (2017a) Research on nutrient pollution load in Lake Taihu, China. Environmental Science and Pollution Research*,* **24**, 17829-17838.

Wang J, Zhou K, Wang X *et al.* (2015a) Atmospheric nitrogen and phosphorous deposition in Hangjiahu area. China Environmental Science*,* **35**, 2754-2763 (in chinese).

Wang R, Balkanski Y, Boucher O, Ciais P, Penuelas J, Tao S (2015b) Significant contribution of combustion-related emissions to the atmospheric phosphorus budget. Nature Geoscience*,* **8**, 48-54.

Wang R, Goll D, Balkanski Y *et al.* (2017b) Global forest carbon uptake due to nitrogen and phosphorus deposition from 1850 to 2100. Global Change Biology*,* **23**, 4854-4872.

Wang X, Yin W, Shan Y, Feng K, Zhu J (2009) Nitrogen and phosphorus input from wet deposition in Taihu Lake region: A case study in Changshu Agro-ecological Experimental Station. Chinese Journal of Applied Ecology*,* **20**, 2487-2492 (in chinese).

Wei D, Li X, Zhao Y, Xu H, Feng H, Yu Z (2018) Analysis of atomspheric nitrogen and phosphorus deposition at Hefei Science Island and its impact on Chaohu Lake. Journal of Hefei University technology*,* **41**, 1259-1266 (in chinese).

Wright IJ, Reich PB, Westoby M *et al.* (2004) The worldwide leaf economics spectrum. Nature*,* **428**, 821-827.

Wu Y, Huang X, Jiang Z, Liu S (2021) Identifying sources of phosphorus in precipitation using phosphate oxygen isotope in a human and monsoon Co-affected embayment. Atmospheric Environment*,* **244**.

Wu Y, Zhang J, Liu S, Jiang Z, Huang X (2018) Aerosol concentrations and atmospheric dry deposition fluxes of nutrients over Daya Bay, South China Sea. Marine Pollution Bulletin*,* **128**, 106-114.

Xing J, Song J, Yuan H *et al.* (2018) Water-soluble nitrogen and phosphorus in aerosols and dry deposition in Jiaozhou Bay, North China: Deposition velocities, origins and biogeochemical implications. Atmospheric Research*,* **207**, 90-99.

Xu F, Gao Y, Dong W, Hao Z, Xu Y (2016) Impact of atmospheric nitrogen and phosphorus wet deposition on nitrogen and phosphorus export and associated water quality: a case study of forest watershed in the red soil area， Southern China. Acta Ecologica Sinica*,* **36**, 6409-6419 (in chinese).

Xu Q, Yang T, Yang X, Ge X (2003) Analysis and prediction of eutrophication for Miyun reservoir. Journal of jilin university, 315-318 (in chinese).

Yang L, Wang M, Wang C (2018) Nitrogen and phosphorous characteristics of canopy rainfall of eight dominant tree species in secondary forests of Shennongjia Mountain. Chinese Journal of Ecology*,* **37**, 1018-1025.

Yu G, Yang C, Liu K, Yang H, Zhang J (2017) Atmospheric deposition of nitrogen and phosphorous in Lake Yangzonghai,Yunnan Province. Journal of Lake Sciences*,* **29**, 1134-1142 (in chinese).

Zhai S, Yang L, Hu W (2009) Atmospheric nitrogen and phosphorus deposition during optimal algal growth period in northern Lake Taihu. Environmental Pollution & Control*,* **31**, 5-10.

Zhang H, Zhu Y, Li F, Chen L (2011) Nutrients in the wet deposition of Shanghai and ecological impacts. Physics and Chemistry of the Earth*,* **36**, 407-410.

Zhang J, Chen SZ, Yu ZG, Wang CS, Wu QM (1999) Factors influencing changes in rainwater composition from urban versus remote regions of the Yellow Sea. Journal of Geophysical Research-Atmospheres*,* **104**, 1631-1644.

Zhang J, Yu Z, Zhang J, Chen N (2000) Wet Deposition (Preciptation) of Major Elements at Two Sites of Northwestern Yellow Sea. Environmental Chemistry*,* **19**, 352-356 (in chinese).

Zhang N, Wei Q (1992) Chemical composition and distribution characteristics of water-soluble ions in atmospheric particles. Arid environmental monitoring, 69-76 (in chinese).

Zhang X, Lin C, Zhou X *et al.* (2019) Concentrations, fluxes, and potential sources of nitrogen and phosphorus species in atmospheric wet deposition of the Lake Qinghai Watershed, China. Science of the Total Environment*,* **682**, 523-531.

Zhou S, Dang N, Zhang H, Wang J, Ye Y (2022) Atmospheric deposition of nitrogen and phosphorus in forest regions in Kaihua County. Journal of Environmental Ecology*,* **4**, 72-80 (in chinese).

Zhou S, Sun Y, Huang T, Zhan J, Wang H, Li Z (2020) Characteristics of nitrogen,phosphorus and dissolved organic matter in atmospheric wet deposition of Zhoucun Reservoir. Water Resources Protection*,* **36**, 52-59.

Zhu M, Cheng X, Kejia Z *et al.* (2022) Atmospheric Deposition Characteristics and Flux of Nitrogen and Phosphorus in Qiandaohu Reservoir, China. Research of Environmental Sciences*,* **35**, 877-886 (in chinese).

Zhu X, Pan Y, Jiao F, Gu D (2015) Analysis of the atmospheric deposition of nitrogen and phosphorus-taking with Beili Lake as an example. Journal of Safety and Environment*,* **15**, 313-316 (in chinese).

Zhu Y, Liu S (2011) Nutrient in atmospheric wet deposition in East China Sea. Environmental Science & Technology*,* **32**, 2724-2731 (in chinese).
